# Generative Active Learning for Molecular Design with REINVENT: Balancing Binding Affinity and Synthetic Accessibility

**DOI:** 10.1021/acs.jctc.6c01017

**Published:** 2026-09-08

**Authors:** Marco Klähn, Hannes H. Loeffler, Shunzhou Wan, Alexey Voronov, Xibei Zhang, Agastya P. Bhati, Peter V. Coveney

**Affiliations:** † Molecular AI, Discovery Sciences, R&D, AstraZeneca, Mölndal 431 83, Sweden; ‡ Centre for Computational Science, Department of Chemistry, 4919 University College London, London WC1H 0AJ, U.K.; § Wadhwani School of Data Science and Artificial Intelligence, Indian Institute of Technology Madras, Chennai 600 036, Tamil Nadu, India; ∥ Advanced Research Computing Centre, University College London, London WC1H 0AJ, U.K.

## Abstract

We present a generative active learning (GAL) framework for molecular design that integrates the generative AI platform REINVENT with physics-based free-energy estimation via ESMACS, specifically addressing synthetic tractability. In a previous study we have shown that iterative generation and optimization of molecules for protein binding affinity can be effectively achieved. For drug discovery, however, molecules need to be synthesized for experimental validation. Molecules with unclear synthetic routes will be de-prioritized regardless of their predicted binding affinity. Here, we address this by incorporating additional synthesizability constraints into the computational design process, framing the problem as a multi-objective optimization task. We show that it is possible to simultaneously optimize candidate molecules for both binding affinity and synthetic accessibility. Our results show that integrating synthesizability prediction into physics-based GAL workflows enables the efficient design of compounds that are at once chemically diverse as well as predicted to be strong binders and synthetically tractable, demonstrating efficient and practical computational drug design.

## Graphical abstract

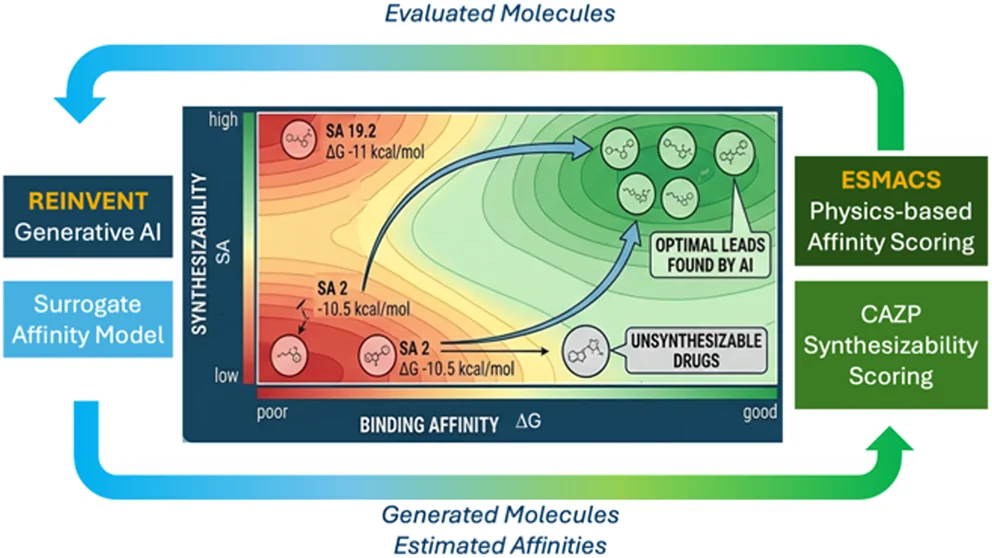

## 1 Introduction

Bringing new therapeutics to the patient has historically been a very challenging and resource-intensive endeavor,^1–3^ highlighting the complexity and inherent risks of drug discovery and development. While failure of a drug candidate in clinical trials presents a major financial risk, improvements in the discovery stage with respect to duration, cost and quality are expected to de-risk the clinical development.

Acceleration efforts using computers have a long tradition in drug discovery, involving quantitative structure-activity relationships (QSAR),^4^ molecular docking^5^ and molecular simulations.^6^ In recent years, advances in machine learning (ML) methods and the availability of larger amounts of data have enabled artificial intelligence (AI) to impact drug discovery and development leading to accelerations in drug design.^7^ In particular, the use of generative AI is leading to exploration of previously untapped chemistry.^8,9^

Generative molecular design in drug discovery^10–12^ uses reinforcement learning,^13,14^ adversarial networks,^15^ autoencoders^16^ and diffusion models^17^ to find optimized molecules with respect to desirable properties, e.g., potency, ADME (absorption, distribution, metabolism, excretion), toxicity, solubility, target specificity and synthetic accessibility. As the chemical space of drug like molecules is expected to exceed exhaustive search by far,^18^ generative AI methods are hoped to explore unseen chemical space and thus to overcome the limits of existing chemical collections. Importantly, generative AI methods may fail to produce meaningful molecules if not properly constrained by physical, chemical and biological considerations and fundamental challenges still persisting have been pointed out.^19^ Grounding AI methods in physics is therefore of utmost importance.^20^ To address this, several studies have combined generative models with more accurate physics-based methods based on molecular dynamics (MD) simulations, such as relative binding free energy (RBFE) methods^21^ or molecular mechanics Poisson-Boltzmann surface area methods (MMPBSA)^22^ which are capable of predicting binding affinities with significantly higher accuracy than traditional docking methods.

Physics-based methods are computationally expensive, but have the potential to be accurate and are independent of a pre-defined applicability domain as in data-driven methods. The latter is of particular importance for generative methods which are hoped to produce novel structures in chemical space, for which generated molecules are out-of-domain of ML models. Hence, active learning (AL) has been shown to be an attractive strategy for integrating such expensive methods into molecular design workflows.^23–25^ AL iteratively selects the most informative candidates from a large pool of generated molecules for evaluation using higher-accuracy methods, e.g., free energy perturbation (FEP) or MMPBSA, and using a surrogate ML model to evaluate the remaining molecules at a much reduced computational cost, improving model performance over multiple AL iterations. Importantly, this strategy aligns naturally with the design–make–test–analyze (DMTA) cycle commonly used in medicinal chemistry.^26^ Over the past several years, a number of studies have explored the combination of AL with RBFE calculations to guide lead optimization.^27–32^ These studies demonstrate the potential of AL to efficiently explore chemical space and identify high-affinity ligands. Practical challenges still remain, including computational cost and the need for experimental validation.

To test if the AL approach can be used effectively in hit identification and lead generation, we have recently described our AL approach combined with a highly precise binding affinity prediction method (ESMACS).^33^ We have found AL to be very effective and efficient in maximizing binding affinity. As important, however, as optimal affinity is for a drug and its potency,^34^ a drug must possess beneficial pharmacokinetics/pharmacodynamics (PK/PD), ADME and toxicity properties. Also, from a very practical point of view, synthetic tractability (often simply “synthesizability”) is of particular importance in small molecule drug discovery projects. A compound that is expected to be difficult or costly to make will be de-prioritized regardless of its otherwise drug-likeness. This is particularly important in modern generative approaches where huge numbers of molecules can be easily “made” *in silico* in short periods of time but may be difficult to actually synthesize.^35^ Synthetic tractability is therefore a substantial bottleneck for drug discovery.

Several approaches have been proposed to estimate synthesizability, ranging from simple heuristic scores^36–38^ to more sophisticated ML models and retrosynthetic planning algorithms.^39,40^ Simple synthesizability scores typically estimate only likelihoods, however, without mechanistic insight to guide synthesis. SA score,^36^ despite its name, assumes synthesizability and can be derived through fragment counting and a “complexity” penalty. However, neither of these are strongly correlated with synthetic tractability and may be biased by data and assumptions used at the time of inception. In contrast, modern retrosynthetic planning tools based on ML methods can propose explicit synthetic routes and evaluate factors such as reaction steps, precursor availability, and route complexity.^41^

In this paper, we extend our previous work^33^ and incorporate synthesizability scores into the AL cycle. We note that we take a rather pragmatic approach on “synthesizability” within the technical limits of current computational approaches. In addition to simple synthesis scores trained on the output of more sophisticated synthesis planners, we also directly apply a retro-synthetic deep neural network approach using AiZynthFinder.^42^ This method is computationally more costly but can also provide synthetic routes, information on availability of starting materials, and number of reaction steps. This synthesis software has been applied extensively in industry and is impacting drug design in numerous projects.^43^ The increased computational cost compared to simpler heuristic scoring methods is negligible in this workflow compared to the far costlier molecular simulations used to derive binding affinities and is therefore not limiting here. The reinforcement learning (RL) approach used by REINVENT also has the advantage that the synthesis planner, and in fact any component scoring function, can be replaced, that is the REINVENT models are not limited by a pre-defined, trained synthesis corpus.^44–46^ Limiting reaction types at the training stage requires later retraining for different chemistry.

By integrating binding affinity prediction with synthesizability assessment within a multi-parameter optimization (MPO) approach, we aim to explore how generative AL balances affinity, diversity, and synthetic tractability of compounds. Understanding this balance will be crucial for translating generative molecular design methods into practical tools for real-world drug discovery.

The literature on applying direct synthesis planning within a complex AL setting is still rather sparse. Filella-Merce and Molina et al. introduce a nested generative AL approach using a variational autoencoder for the generative model.^47^ The inner loop optimizes chemoinformatic-derived properties, including the SA score, while the outer loop executes docking with only the molecules with favorable SA score. Poses of the outer loop, after filtering and selection, are further refined through Monte Carlo simulations. The authors have synthesized nine candidates and found weak to good potency for the CDK2 target in a molecular assay. For KRAS, only absolute binding free energy (ABFE) simulations were provided.^47^ Antoniuk et al. show how a genetic algorithm (JANUS) in connection with a message-passing neural network (MPNN) surrogate model can be used within an AL loop.^48^ Interestingly, the surrogate is trained on density functional theory derived thermodynamic stability data, as a proxy for potentially stable molecules. Experimental validation was not attempted. In our contribution, we aim to show that a transformer-based generative AL approach with explicit retro-synthesis planning can be applied successfully.

## 2 Methods

In this section, we present the set of methods which together comprise the capabilities we have assembled to enable us to perform combined optimization of both binding affinities and synthesizability of molecules proposed by REINVENT.

### 2.1 Reinvent

REINVENT4 ^49^ is a well-established generative AI software environment for *de novo* generation of small molecules and peptides. Since its inception in 2017,^50^ REINVENT^49–51^ has been developed into a well-benchmarked generative molecular design engine and a *de-facto* industry standard;^52,53^ it is also frequently used in academia. The current version, REINVENT4 is available in a Github repository.^54^ The code is used in production internally at AstraZeneca in most drug discovery programs.^55–58^ Extensive benchmarks have been run for REINVENT,^59–65^ where some benchmarks have also been structured around REINVENT.^60,65^ Most importantly, REINVENT has been experimentally validated in several publications.^57,58,66–70,88^ REINVENT is therefore used here as an established molecular-generation framework rather than as a novel methodological contribution to this work.

In this work we use the recurrent neural network (RNN) based, classical REINVENT prior which generates new molecules entirely from scratch, i.e., no molecule templates are invoked. REINVENT uses reinforcement learning (RL) to drive the agent, a copy of the RNN model, towards generating molecules with desired properties with higher probabilities. For this the RL reward function is informed by an aggregation of individual scores where each component is a function to assess (“score”) the quality of a molecule. These scores can be physico-chemical properties, regression and binary classifiers, e.g., for affinity prediction, physics-based models like docking or molecular dynamics workflows, synthesizability estimation and so on. In fact, the modular software system allows any scoring component to be implemented in code. The user can choose from a set of transformation functions to ensure each scoring component is in the range between 0 and 1. The aggregation is typically done through a weighted geometric mean (i.e., a product of components) to ensure that the RL algorithm does not optimize for a subset of the components only as may be the case with the arithmetic mean (sum).

### 2.2 Synthesizability Scores

For this work we have assessed various synthesizability scores for their impact on optimization with AL. Specifically, we looked into a full retro-synthesis approach with AiZynthFinder 4.0 ^42^ (dubbed “CAZP” for the remainder of the text), RAScore^71^ (“RA”) which was trained on predicting routes from 200,000 ChEMBL compounds from AiZynthFinder and thus limited to that chemical space, and the RetroGNN M1Score,^72^ a message-passing graph neural network^73^ which was trained on the M1 synthesis planner (“M1”) from Molecule.one with USPTO data. The planner considers both the probability of finding a route and a heuristic to estimate the cost of intermediates. We have also carried out REINVENT runs using both the M1Score and the RAScore (“M1 + RA”) together. As both RA and M1 are simple models only providing a single score for how feasible the synthesis of a molecule may be, they perform substantially faster than the retrosynthetic approach taken by AiZynthFinder. AiZynthFinder, in contrast, provides rich information on actual synthetic routes as trees of possible routes starting from “stock” compounds. As mentioned, the increased computational cost of AiZynthFinder compared to other scoring methods is not a concern in the context of our workflow, as it contains computationally much costlier components that involve molecular simulations.

For AiZynthFinder we used the public USPTO models and Zinc stock database.^74^ The CAZP score for REINVENT was computed as the product of the AiZynthFinder scores for building block availability (1.0 if in Zinc stock, 0.1 otherwise), reaction class membership (no class constraints were used in this work, so always 1.0) and the reaction step coefficient (0.9) raised to the power of the number of predicted reaction steps. AiZynthFinder was allowed to suggest reactions up to 5 reaction steps.

### 2.3 ESMACS

Binding free energies were estimated using the ESMACS (enhanced sampling of molecular dynamics with approximation of continuum solvent) protocol.^75^ This ensemble-based MMPBSA approach allows for the calculation of binding affinities with reliable uncertainty estimates, facilitating a deeper insight into the structural and energetic nature of ligand–protein binding. As an end-point method, the free energy is derived from the difference between the bound complex and its separated components. Here conformations of the complex, protein and compound are all extracted from simulation of the complex. Although this specific extraction protocol and the broader approximations of the approach commonly lead to a characteristic overestimation of absolute binding free energies, the relative trends remain highly reliable. This approach is particularly effective for hit identification, where achieving an accurate ranking of binding affinities is the primary objective for selecting candidate compounds for downstream analysis.

To prepare the REINVENT-generated molecules (Section 2.1) for simulation, we first assigned appropriate protonation states at pH 7.4 using FixpKa 2.1.3.0 [Cadence Molecular Sciences, Santa Fe, NM].^74^ We then expanded the conformational space for each compound by generating a library of up to 200 conformers per molecule with OMEGA 4.1.2.0.^76^ This maximum limit represents the default software parameter, which has been demonstrated to balance exhaustive conformational space sampling with virtual screening efficiency.^77^ These structures were subsequently docked into the target proteins, 3CL^pro^ (PDB: 6W63) and TNKS2 (PDB: 4UI5), using FRED v4.1.1.0 ^51^ under its default settings, including resolution parameters of 1.0 Å for translational step size and 1.5 Å for rotational step size, and Chemgauss4 scoring. Literature benchmarking confirms that under these default settings, FRED achieves high-fidelity pose accuracy, successfully placing over 70% of targets within a 2.0 Å RMSD of experimental structures.^78^ For each compound, the pose associated with the highest Chemgauss4 score was selected as the starting point for all subsequent ESMACS simulations.

The crystal structures of the proteins were prepared using the OpenEye SPRUCE tool under its default parameter settings. This process accounted for biologically relevant unit extraction, loop and missing residue modelling, tautomer and protonation state enumeration, and hydrogen bond network optimization.

Preparation and setup of the ESMACS simulations were managed via the binding affinity calculator (BAC).^79^ We employed AmberTools23 ^80^ to build the systems and perform the final free energy evaluations. Small molecules were parameterized using the general Amber force field 2 (GAFF2) with atomic partial charges derived from the AM1-BCC scheme. The Amber ff14SB force field was used for the proteins, and TIP3P for explicit water molecules. The protonation states of the protein residues were assigned using the “reduce” module of AmberTools. All crystallographic water molecules were kept in the starting structures. Each complex was immersed in an orthorhombic box of TIP3P water, ensuring a 10 Å buffer between the solute and the box boundary, with counterions introduced to electrostatically neutralize the systems.

To accommodate the high-throughput requirements of this screening project, we adapted the standard ESMACS protocol, typically consisting of 25 replicas, into a more computationally efficient “coarse-grained” version. By employing an ensemble of 10 replicas and a reduced 10 Å solvent buffer (down from the standard 14 Å), we significantly accelerated the evaluation of the large compound library. While this modification involves a slight trade-off in precision, it is optimized for ranking vast numbers of ligands and well-suited for high-throughput virtual screening. NAMD3 ^81^ was used as the MD engine for all of the minimization, equilibration and production runs. For each individual simulation, energy minimizations were first performed with heavy protein atoms restrained at their initial positions. Systems were then heated to and maintained at 300 K over 60 ps with initial velocities generated from independent Maxwell–Boltzmann distributions. Finally, 4 ns production runs were performed for each replica. Exploiting the Frontier exascale supercomputer, we executed all simulations concurrently. With a performance of 150 ns/day per AMD Instinct MI250X GPU, the entire batch of 100 compounds for each GAL iteration was completed in just 50 min of wall-clock time.

The ESMACS simulations comprised the primary computational expenditure, consuming over 90% of the overall computational resources dedicated to this study. In total, we simulated ∼5,500 compounds using 10 independent replicas per molecule with a 4 ns production run per replica, requiring approximately 46,000 GPU hours on the Frontier supercomputer.

### 2.4 Active Learning

We have described our active learning (AL) workflow in detail recently.^33^ To bootstrap the AL cycle, in the case of 3CL^pro^, we computed free energies of binding with ESMACS for the top 10,000 molecules found via surrogate docking. The data were used to train a predictive model (surrogate model) for use as a scoring component in REINVENT. We used ChemProp^73^ because deep neural networks are particularly useful for large numbers of molecules. In the case of TNKS2, 27 compounds with known measured affinities were used to train a random forest model that was used subsequently to generate 10,000 molecules with REINVENT. The surrogate model was then built in the same way as for 3CL^pro^ using ChemProp training on the 10,000 REINVENT generated molecules.

ChemProp models were created using 5 iterations and 5 ensembles in cross-validation, using an 80/10/10 split for training, test and validation sets. Normalized RDKit 2D features were applied to augment the MPNN graph-based fingerprint. In each GAL step, the surrogate model was retrained from the accumulating data set starting from the model of the previous step. Only the single best model, based on test RMSE, was used for REINVENT. During GAL, the ChemProp surrogate model was retrained at each iteration with acquired compounds and ESMACS binding affinities. The quality of the retrained surrogate model was monitored throughout GAL by comparing estimated binding affinities with ESMACS results.

Together with QED,^82^ control of the number of stereocenters and a filter for unwanted structural motifs, the surrogate model was interrogated to generate new compounds for the next AL cycle. The synthesizability scores (see above) were added directly to the REINVENT RL training. In the case of CAZP we ran in two separate stages, where the first stage used only QED, stereocenter control and structure filtering. CAZP was only phased-in for the second stage as it was found that convergence was otherwise difficult to achieve. We added the synthesizability scores either only at the last AL cycle or in all AL cycles to assess how this impacts AL optimization.

Molecules with the highest scores (greedy acquisition) in each cluster, as described earlier,^33^ were collected in a batch of 100 compounds and subjected to free energy simulations with ESMACS to initiate the next AL cycle.

The entire GAL workflow is shown in Figure 1 and is described in its caption.

**Figure 1:**
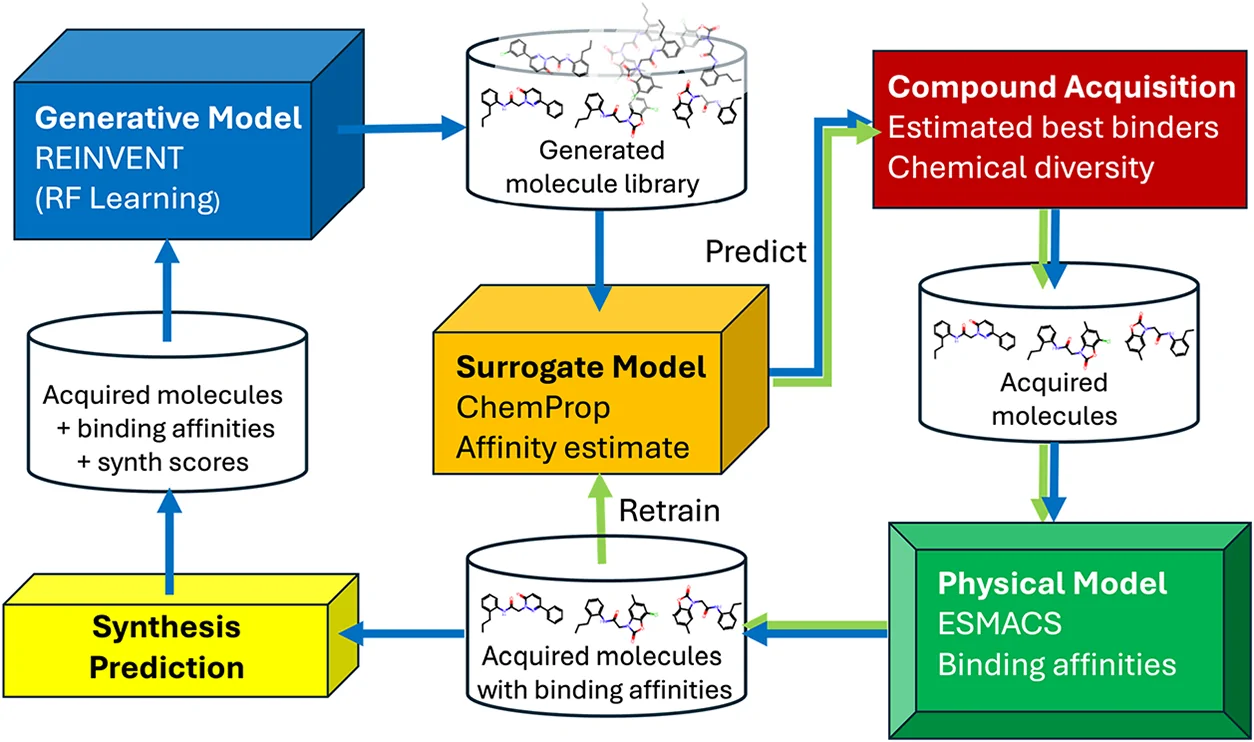
GAL workflow with ML components (3D boxes) and physical model. REINVENT generates a molecule library for which the ChemProp surrogate model estimates binding affinities. For this library, molecules are selected with strong estimated binding affinities, while taking into account chemical diversity. Binding affinities of acquired molecules are derived with the physical ESMACS method. These compounds with derived affinities are used to update the surrogate model as preparation for the next iteration. Synthesizability scores are added for acquired molecules and combined with binding affinities to train REINVENT with reinforcement learning. The GAL process therefore consists of two integrated optimization loops for REINVENT (blue arrows) and the surrogate model (green).

### 2.5 Test Targets

Tankyrase-2 (gene TNKS2) was taken as a test case from a large-scale binding free energy calculation benchmark data set. TNKS2 is oncogenic and regulates various cellular processes, such as telomere maintenance, mitosis, and glucose metabolism. It was chosen to study performance of GAL for a target with a more closed and confined binding pocket, compared to 3CL^pro^. It was also selected as a suitable test-case for a target for which 27 experimentally confirmed ligands from a congeneric series were available. These were used as seed structures to initialize GAL (see above) in order to see if binding affinities could be improved further and how far these generated structures would diverge structurally from the original ligands. Preparation of TNKS2 for simulations is described above in Section 2.3.

C30 endopeptidase, usually referred to as 3CL^pro^ or M^pro^, is the main protease in coronaviruses and plays a vital role in the lifecycle of SARS-CoV-2 (severe acute respiratory syndrome-coronavirus-2) which is the cause of COVID-19 (coronavirus disease 2019). COVID-19 led the WHO to declare a worldwide epidemic from 30 January 2020 to 5 May 2023 after the first case was confirmed in November 2019. Hundreds of millions of cases have been confirmed since then and several million deaths have been directly linked to the virus. The virus and its various mutations are still a global threat to health. Several medications such as remdesivir (Veklury), nirmatrelvir/ritonavir (Paxlovid), baricitinib (Olumiant), tocilizumab (Actemra), and molnupiravir (Lagevrio) are available to treat various stages and forms of COVID-19. However, there is still a need to find new active compounds to deal with the continuously mutating virus and future related diseases. Preparation of 3CL^pro^ for simulations is described in Section 2.3.

## 3 Results

### 3.1 Method Validation

#### 3.1.1 ESMACS Validation for TNKS2 and 3CL^pro^

The ranking performance of ESMACS and the docking protocol were evaluated in our preceding work, using a dataset of 1014 TNKS2 inhibitors with experimentally measured IC_50_ values from BindingDB. For this system, docking by itself exhibited negligible ranking capability as expected (Spearman’s ρ = 0.08), indicating that the docking protocol was indeed unsuitable for identifying strong TNKS2 ligand binders. In contrast, ESMACS achieved moderate but clearly superior ranking performance (ρ = 0.33). It has previously been shown that ESMACS provides accurate rankings.^83–85^ This supports the use of ESMACS as a higher-fidelity oracle compared to docking for TNKS2, even under adverse data conditions.

As mentioned in our preceding work, for 3CL^pro^, direct validation against experimental affinity data was not feasible due to inconsistencies arising from rapid viral mutation and heterogeneous assay conditions. Instead, the effectiveness of ESMACS-driven AL was assessed indirectly by comparing its greedy acquisition strategy, as described above, against random compound selection. In both TNKS2 and 3CL^pro^ systems, the ESMACS-guided cluster-greedy acquisition consistently identified stronger binders more efficiently than random selection, as evidenced by systematic shifts toward lower free energy values in the resulting distributions.

#### 3.1.2 Validation of Single Objective AL Approach

In the preceding work, we validated the effectiveness of AL for single-objective optimization of binding affinity by introducing the GAL (generative active learning) protocol, which couples REINVENT-based molecular generation with physics-based binding free energy estimation using ESMACS as an oracle. Applied to the two mechanistically distinct targets, TNKS2 and 3CL^pro^, GAL consistently generated ligands predicted to have high binding affinities that were both structurally diverse and substantially different from the compounds used to initialize surrogate model training.

For TNKS2, the narrowly confined binding pocket led to a well-defined binding mode, enabling surrogate models with higher accuracy and rapid convergence of AL within 3–4 iterations. For the more challenging 3CL^pro^ target, characterized by a larger and more flexible binding site with multiple binding modes, surrogate models exhibited reduced accuracy, requiring additional (5–7) iterations to achieve convergence. Despite these differences, GAL successfully optimized the binding affinity for both systems, demonstrating robustness of the AL framework across targets with distinct binding landscapes.

### 3.2 GAL Performance for Test Case TNKS2

#### 3.2.1 Performance of GAL-Surrogate Model

We first assessed the quality of the surrogate model for the TNKS2 target during GAL in the absence of any synthesizability constraints in Figure S1 and with addition of synthesizability scores (SYNS) in Figure S2. The performance of the two best surrogate models, M1 + RA and CAZP, is shown in Figure 2. In these figures we compare binding affinities, Δ*G*_bind_, of generated ligands to TNKS2, as estimated by the surrogate model, with affinities derived from the ESMACS oracle.

**Figure 2:**
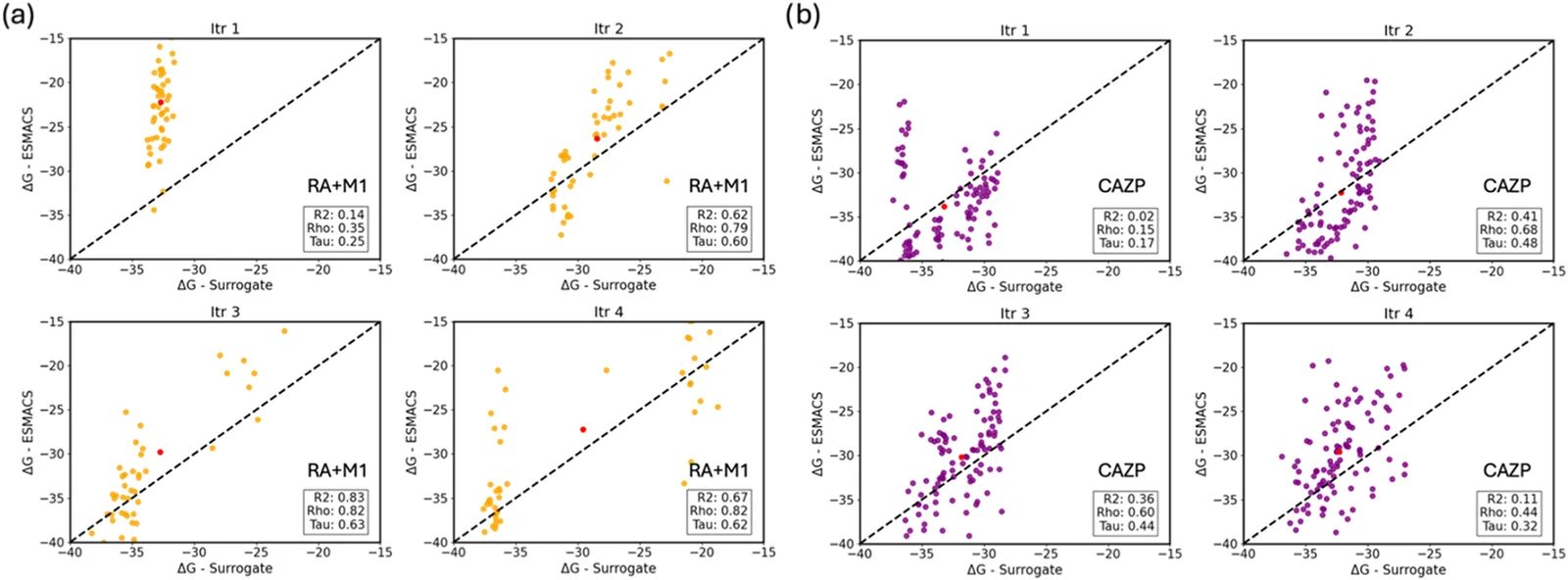
Comparison of Δ*G* predicted by the surrogate model with predictions from the ESMACS oracle for different iteration steps of the GAL for TNKS2 using SYNSs RA + M1 (a) and CAZP (b) during the GAL. The *R* ^2^ coefficient, Spearman’s ρ, and Kendall’s τ are given for individual iterations. Average values of Δ*G* from surrogate and ESMACS are indicated with a red dot. All energies are given kcal/mol. Surrogate performance without SYNS and for SYNS M1 and RA are shown in Figures S1–S2.

The observed dynamic range of predicted binding free energies from the surrogate model was generally narrow, especially during the first iteration steps. This is expected, as only the best-performing molecules according to the surrogate predictions were selected and forwarded to the ESMACS oracle for explicit evaluation.

The surrogate model demonstrated a reasonable ability to rank compounds according to their binding free energies as later derived by the ESMACS oracle when no SYNS were used. In particular, the ranking performance, quantified using Spearman’s rank correlation coefficient, reached ρ = 0.27 in the first GAL iteration and improved to ρ = 0.51 by iteration three. This indicates that the surrogate model was able to capture a progressively stronger ordering of candidate molecules as more oracle data became available. Inspection of the affinity comparison plots also revealed an increasing number of data points in the lower left region over successive iterations. These points correspond to true positives, i.e., molecules that were predicted by the surrogate to be strong binders and were subsequently confirmed by ESMACS. In case of RA + M1, the ratio of true positives in each batch was found to increase from 0.28 to 0.64 during the first three iterations, when true positives are defined as compounds with oracle and surrogate predicted affinities below their respective average values as indicated by the red dot in Figure 2. For CAZP, true positive ratios were found to vary more consistently between 0.35 and 0.41 throughout GAL. The accumulation of such points over iterations reflects the improving capacity of the surrogate model to identify promising candidates within the constrained selection regime imposed by GAL.

We next evaluated the impact of incorporating different SYNS into the GAL workflow on surrogate model performance. When SYNS based on RA + M1 was applied, the surrogate model exhibited strong ranking power, with Spearman correlation coefficients in the range ρ = 0.79–0.81 for iterations two through four. Similarly, the CAZP-SYNS led to moderate to strong ranking performance, with ρ values between 0.44 and 0.68 over the same iterations. In contrast, surrogate models trained under synthesis constraints based solely on M1 or RA performed poorly, yielding Spearman correlations below 0.14 and 0.24, respectively. Despite the weak ranking performance observed for M1 and RA when considered individually, a substantial number of true positives were still identified when using M1 and CAZP-based SYNSs.

Overall, the observed surrogate model performance provides grounds for cautious optimism. In our previous work, we demonstrated that even relatively weak surrogate models were sufficient to guide GAL toward successively improved molecules in terms of binding affinity. As we will show later, the results here are consistent with that observation and suggest that, despite variability in ranking quality, the surrogate models retain sufficient predictive signal to drive effective GAL.

#### 3.2.2 Binding Affinity Distributions of Generated Compounds

In the next step we analyzed the binding affinities of the generated molecules. Figure 3 shows the distributions of binding free energies (Δ*G*) for all compounds evaluated with the ESMACS oracle after each GAL iteration, both without synthesizability scoring and with different synthesizability scoring schemes applied. These distributions provide a direct view of how GAL shapes the population of evaluated compounds over successive iterations.

**Figure 3:**
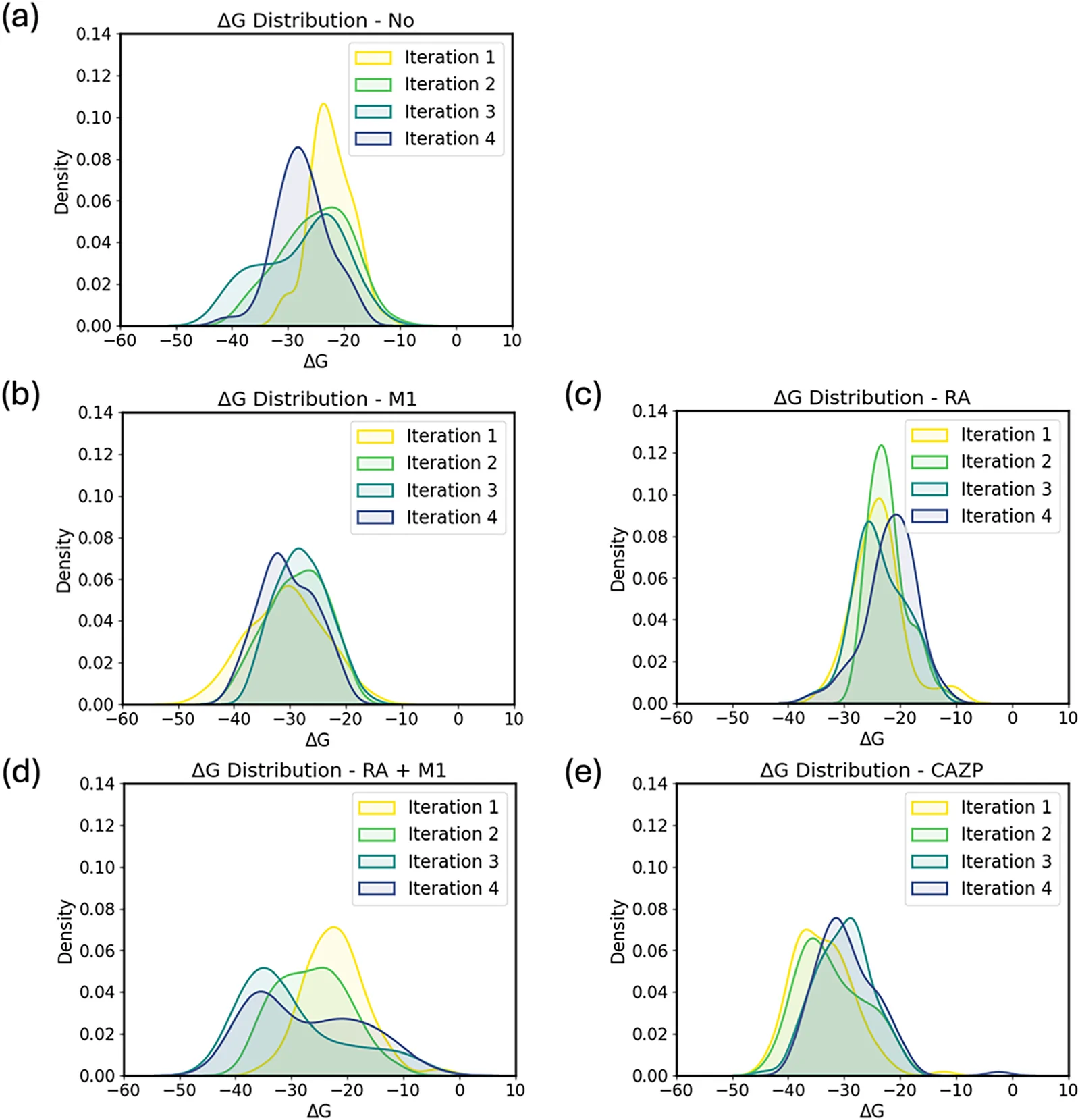
Distribution of Δ*G*_bind_ of compounds evaluated with ESMACS after each AL iteration, without synthesizability score (a) and using synthesizability scoring with M1 (b), RA (c), RA + M1 (d), and CAZP (e) methods. Energies are given in kcal/mol.

In the absence of any SYNS (Figure 3a), the maximum of the binding affinity distribution progressively shifted toward stronger binding as GAL proceeds. Of particular interest is the low-Δ*G* tail of the distribution, corresponding to the strongest binders and representing the primary optimization objective. This high-affinity tail improved steadily up to iteration three, indicating successful enrichment of strong binders. In iteration four, however, the tail recedes slightly, suggesting a degree of convergence or reduced exploration of higher-affinity regions at later stages.

When synthesizability scoring based on RA alone was applied (Figure 3c), performance deteriorated substantially. In this case, GAL failed to identify compounds with binding free energies below −40 kcal/mol, in contrast to values <−45 kcal/mol observed without any synthesizability constraint. This indicates that the RA score imposes a strong restriction on the accessible chemical space that negatively impacts the discovery of high-affinity binders.

In contrast, the combined RA + M1-SYNS (Figure 3d) performed notably well. A pronounced high-affinity tail extending to values below −45 kcal/mol was observed, comparable to the unconstrained case. The distributions suggest rapid improvement during early iterations, with little difference between iterations three and four, indicating apparent convergence of GAL under this scoring scheme.

The M1-SYNS and CAZP-SYNS (Figure 3b,e, respectively) also performed well after a single GAL iteration and yielded binding affinity distributions comparable to those obtained with RA + M1. However, in subsequent iterations the distribution maxima shifted toward weaker binding affinities. In the case of M1, this trend partially reversed in the final iteration, where the distribution again shifted toward stronger binding, suggesting renewed enrichment of higher-affinity compounds.

Overall, the expectation was that introducing synthesizability constraints would lead to systematically weaker binding affinities due to the more restricted chemical space imposed by the dual optimization objectives of affinity and synthesizability. However, with the notable exception of the RA score, the binding affinity distributions were not strongly negatively affected by the inclusion of synthesizability scoring. In several cases, high-affinity tails comparable to the unconstrained GAL setup were retained, demonstrating that effective optimization of binding affinity certainly remains possible even under synthesizability constraints.

#### 3.2.3 Distribution of Synthesizability Scores of Generated Compounds

We have seen that good binders could still be found despite the dual optimization goal. In the following we seek to confirm whether the binders found also achieve high SYNS. Figure S3 shows the distributions of SYNS for TNKS2 compounds that were evaluated with the ESMACS oracle after each GAL iteration, using the M1, RA, RA + M1, and CAZP scoring methods. This analysis focuses on how effectively GAL optimizes synthesizability alongside binding affinity.

For the M1-, RA-, and CAZP-SYNS (Figure S3a,b,d), the distributions indicate that synthesizability was optimized efficiently during GAL. In the case of RA and CAZP, nearly all evaluated compounds achieved the maximum possible score of 1. For M1, the majority of compounds attained high scores in the range of 0.9–1.0. These results suggest that GAL was able to rapidly identify and retain compounds with high synthesizability according to these scoring functions. The combined RA + M1-SYNS (Figure S3c) exhibits broader score distributions, particularly GAL iterations 2 and onwards, even though after iteration 4, the majority of compounds were found with a score >0.7. This behavior indicates a moderate increase in difficulty in simultaneously optimizing binding affinity and the combined synthesizability objective. A likely explanation is that merging RA and M1 into a single constraint creates a more complex optimization landscape, making it harder for the generative model to satisfy both criteria at the same time.

Overall, these results indicate that GAL had little difficulty identifying compounds with high SYNS while simultaneously optimizing for strong binding affinity. Except for the combined RA + M1 case, SYNS converged rapidly toward their upper limits, demonstrating that the inclusion of synthesizability constraints does not, in general, impede the ability of the GAL framework to generate synthetically favorable candidates, at least as estimated according to these scoring approaches.

#### 3.2.4 Chemical Diversity of the Strongest Binding Compounds

The most relevant compounds generated in this process are those with high binding affinity. Therefore, we proceeded with the analysis prioritizing those compounds. To assess their chemical diversity, we analyzed pairwise Tanimoto similarity distributions for the cumulatively best 25 compounds evaluated with ESMACS for TNKS2. Tanimoto similarities of compounds were measured using their Morgan fingerprints, with a radius of 2 and 2048 bits, as implemented in RDKit.^86^ For each iteration, the set of top binders was constructed by pooling compounds from this and all previous iterations. Chemical diversity was quantified by computing Tanimoto similarities between all molecule pairs within this set. The results are shown in Figure 4.

**Figure 4:**
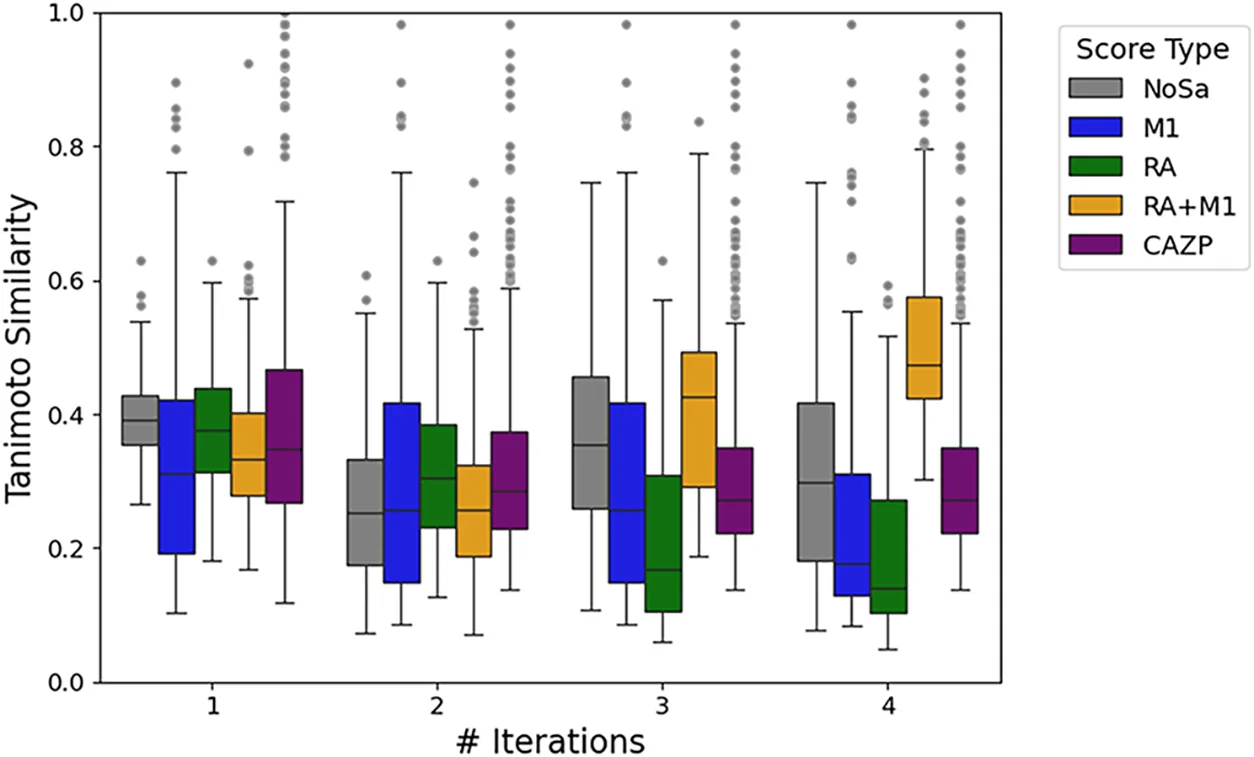
Tanimoto similarity distributions of molecule pairs, taken from the 25 strongest binding compounds of accumulatively generated molecules evaluated with ESMACS and evaluated after each GAL iteration for TNKS2, with M1, RA, RA + M1, and CAZP synthesizability scoring and without SYNS.

The M1 and RA scoring schemes exhibited the lowest median pairwise Morgan fingerprint similarities among the top binders. After iteration four, median similarities were approximately 0.20 for M1 and 0.15 for RA, compared with approximately 0.30 in the absence of synthesizability scoring. Although all three values correspond to generally low pairwise structural similarity, the lower median similarities observed for M1 and RA indicate reduced redundancy within the set of highest-affinity compounds and are consistent with broader exploration of chemical space.

The CAZP scoring scheme resulted in a median Tanimoto similarity of approximately 0.29, comparable to compound similarity observed without synthesizability scoring. This suggests that CAZP neither strongly enhanced nor substantially restricted chemical diversity among the top binders.

In contrast, limited chemical diversity was observed when the combined RA + M1-SYNS was used. In this case, the median Tanimoto similarity increased to approximately 0.48 and continued to rise over successive iterations. This trend indicates progressive enrichment of structurally similar compounds among the strongest binders, consistent with a narrowing of chemical space exploration under the combined constraint.

Interestingly, the RA scoring scheme shows an increase in chemical diversity of the strongest binders with increasing iteration number. In contrast, the diversity associated with CAZP and, to a lesser extent, M1 remains relatively constant across iterations. This behavior is consistent with earlier observations that the strongest binders for CAZP and M1 were predominantly identified after the first GAL iteration, after which the Δ*G* distributions shifted toward less favorable binding energies.

Overall, these results suggest that synthesizability scoring can influence the balance between exploitation and diversity among top-ranked binders, with distinct effects depending on the specific scoring method employed.

#### 3.2.5 Binding Affinity Distributions of the Strongest Binding Compounds

To directly evaluate the success of GAL in identifying high-affinity binders, we analyzed the Δ*G*_bind_-distributions of the 25 strongest binding compounds identified cumulatively across GAL iterations for TNKS2. An average standard error of 0.8 kcal/mol for Δ*G*_bind_ was derived from affinity variations across 20 simulation replicas. The results are shown in Figure 5.

**Figure 5:**
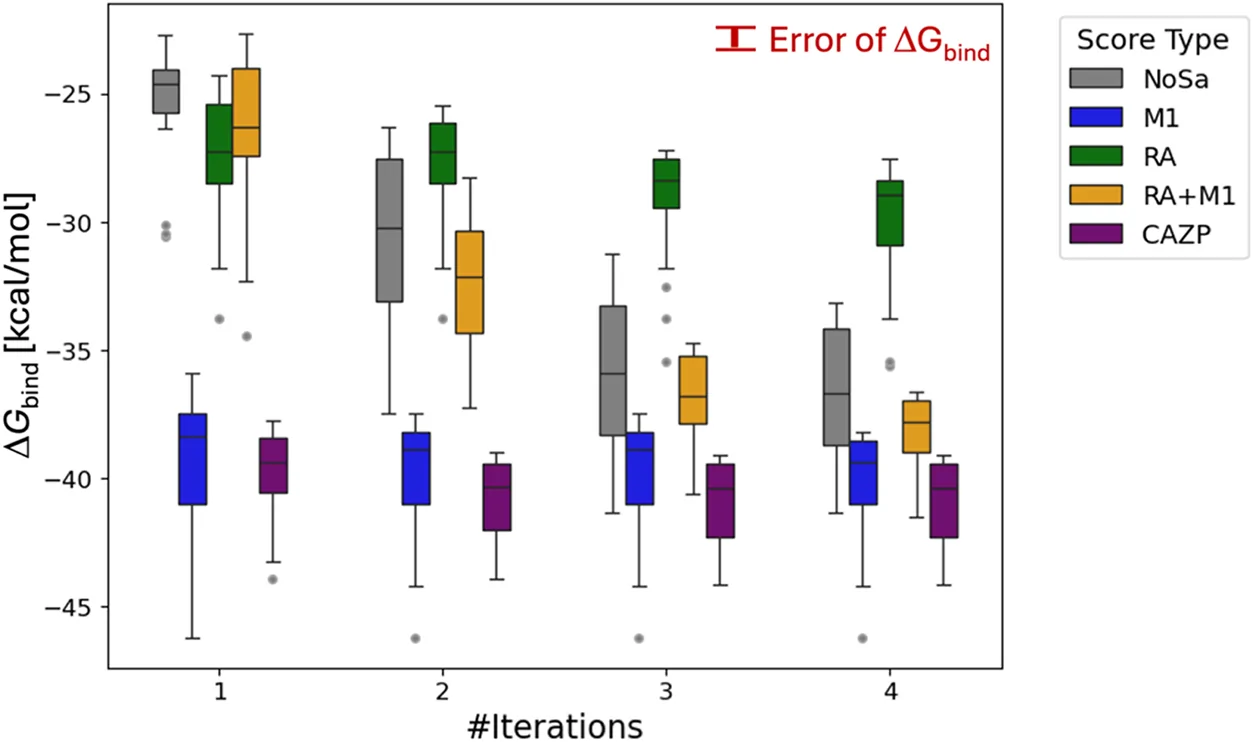
Binding free energy distribution of molecules taken from 25 strongest binding compounds of accumulatively generated compounds evaluated with ESMACS and evaluated after each GAL iteration for TNKS2, with M1, RA, RA + M1, and CAZP synthesizability scoring and without SYNS. The standard error of Δ*G*_bind_ = 0.8 kcal/mol is indicated for comparison in the top right corner.

In the absence of synthesizability scoring, the binding affinity of the strongest compounds improved consistently with each GAL iteration. This trend indicates that new high-affinity binders were discovered at every step of the optimization process. After four iterations, a median Δ*G*_bind_ of −37 kcal/mol was reached, contributing to an increasingly favorable pool of top-ranked compounds.

Among the synthesizability scoring methods, the strongest binders were identified when using M1 and CAZP. For M1, a median Δ*G*_bind_ of −38 kcal/mol was observed, while CAZP achieved a median Δ*G*_bind_ of −40 kcal/mol. In both cases, Δ*G*_bind_ distributions extended to about −44 kcal/mol, with an outlier reaching −46 kcal/mol in the case of M1. Notably, for both M1 and CAZP, no further improvement in binding affinity was observed in subsequent GAL iterations, indicating that the strongest binders were already identified after the first iteration.

In contrast, when the combined RA + M1-SYNS was applied, binding affinity continued to improve over successive GAL iterations, reaching a median Δ*G*_bind_ value of −38 kcal/mol after iteration four. This suggests that additional iterations might have led to binders with affinities comparable to those observed for M1 and CAZP alone.

The use of the RA-SYNS performed poorly in terms of binding affinity optimization. In this case, GAL failed to identify strong binders, achieving only a median Δ*G*_bind_ of −28 kcal/mol after four iterations.

Overall, these results demonstrate that GAL was successful in generating compounds with both diverse chemical structures and strong binding affinities. Importantly, the addition of synthesizability scoring did not generally compromise the ability of GAL to identify high-affinity binders. The only notable exception was the RA scoring scheme, which substantially limited the discovery of strong binders.

In addition to the preceding quantitative analysis, the chemical structures of the strongest binders were examined to gain qualitative insight into the chemical spaces explored by GAL under different synthesizability scoring schemes. Figures S4–S8 show the 25 strongest binding compounds identified at the end of GAL without SYNS and with M1, RA, M1 + RA, and CAZP. The 5 strongest binders without SYNS and with CAZP and M1 are shown in Figure 6. The compounds are depicted in order of decreasing binding affinity as estimated by ESMACS.

**Figure 6:**
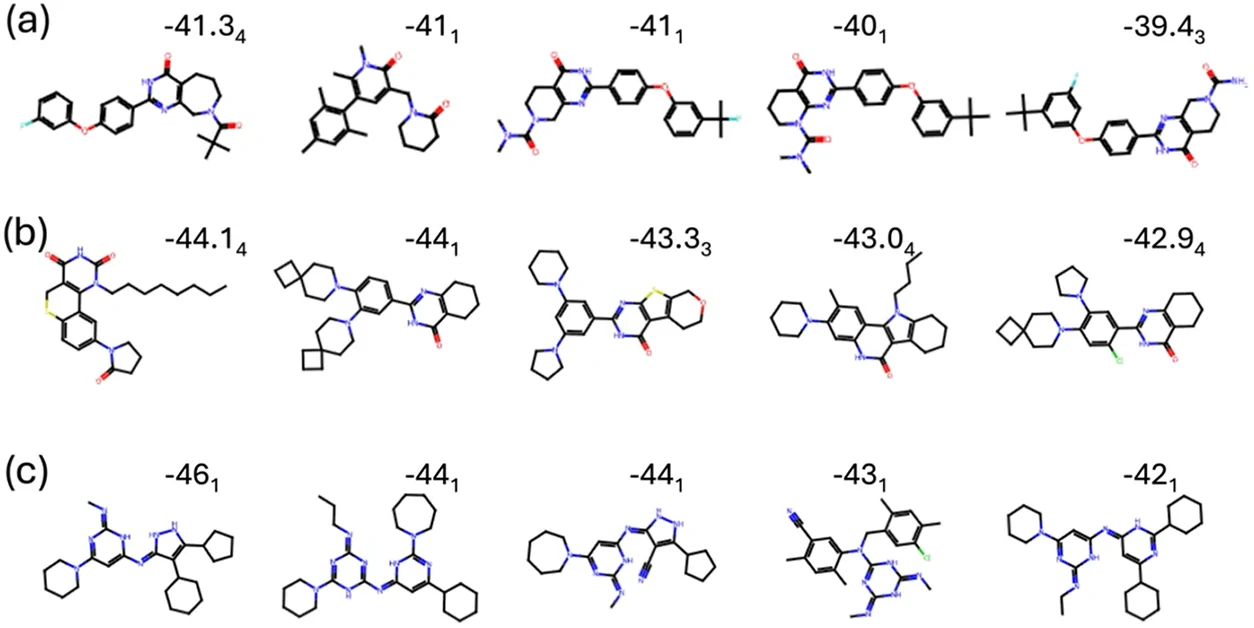
Five strongest binding compounds, depicted in order of decreasing affinity strength according to ESMACS for TNKS2, found without SYNS (a), with CAZP (b) and M1 (c) at the end of GAL. Numbers are binding free energies of compounds and are given in kcal/mol with standard error as index. The 25 best binding compounds are shown in Figures S4–S8.

Comparison of the molecular structures obtained with CAZP and M1 scoring reveals substantial chemical variation between the two sets of top-ranked compounds. The differences observed between CAZP- and M1-derived binders are larger than those typically seen when comparing compounds generated within the same GAL setup. This suggests that GAL, guided by different synthesizability scoring functions, converges towards distinct regions of chemical space.

In case of CAZP, compounds were generated around a flat heteroatomic bicyclic core with two or three ring-nitrogen. In most cases, four bonds farther, a secondary amide can be found with a hydrophobic benzene ring in between, with other lipophilic moieties attached to rings of the compound.

At the core of M1-generated compounds are typically found two heteroatomic 6-membered rings with two substituted nitrogen atoms each, linked via another nitrogen. To this core are typically two or three further hydrophobic rings attached.

Despite this divergence between scoring schemes, a considerable degree of chemical variation is present within each set of top binders. For both CAZP and M1, multiple scaffolds are observed among the strongest binders, as well as series of compounds sharing a common scaffold but differing in peripheral substituents. This indicates that, even at the level of the strongest binders, GAL-generated compounds were not limited to a single chemotype.

Diversity of scaffolds was investigated further, with Murcko scaffolds being extracted for all compounds and compared with each other.^87^ For CAZP all scaffolds were unique, for M1 and RA only two and one duplicate scaffolds were found, respectively, and for RA + M1 one scaffold was found four times and one twice in the ensemble. Average Tanimoto similarities of Murcko scaffolds were found to be <0.35 in all cases, except in the case of RA + M1 where a relatively large value of 0.48 was found in line with observations made in the previous section (Figure 4). Generated scaffolds of the strongest binders often share common motives that are connected in different ways and could belong to the same scaffold family as can be seen in Figures S4–S8.

A qualitative difference in molecular shape is also apparent between the compounds generated using the two scoring schemes M1 and CAZP. Compounds identified using CAZP scores tend to exhibit more linear molecular structures, whereas those generated with M1 scoring display a broader range of shapes and connectivities. This observation is consistent with the notion that different SYNS impose distinct biases on the generative process, influencing not only binding affinity but also the overall structural characteristics of the resulting compounds. Moreover, considering the highly non-deterministic nature of the GAL process, repeated GAL runs using the same scoring scheme will not necessarily converge to the same chemical spaces.

#### 3.2.6 Exploration of Chemical Space during GAL

To further analyze how GAL explored chemical space under different synthesizability constraints, we examined the chemical space traversed during the optimization process using t-SNE projections of Morgan fingerprints. Figure 7 shows the first two t-SNE components for the 25 strongest binders for TNKS2, both with synthesizability scoring applied throughout GAL and without synthesizability scoring (No SA).

**Figure 7:**
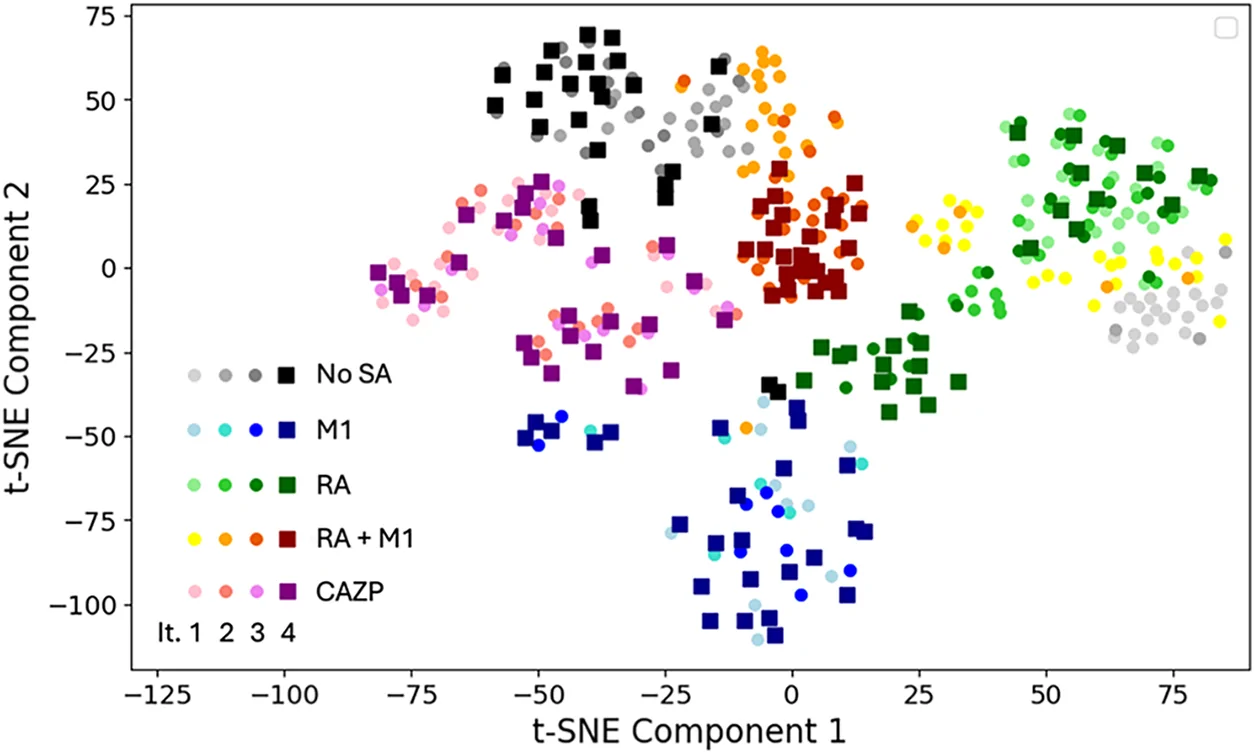
First two t-SNE components of Morgan fingerprints of the 25 strongest binding molecules to TNKS2 of accumulatively generated compounds after each GAL iteration, where synthesizability scoring has been used throughout GAL and without SYNS (No SA).

Across all GAL runs, the use of different synthesizability scoring schemes led to convergence toward distinct regions of chemical space. These regions are also clearly separated from the chemical space sampled in the absence of synthesizability scoring. This indicates that the choice of SYNS has a strong influence on the regions of chemical space being explored and ultimately exploited during GAL.

For the combined RA + M1-SYNS, the chemical space explored during the first two GAL iterations largely overlaps with that observed in the absence of synthesizability scoring. In later iterations, however, iterations three and four showed convergence toward a more compact and distinct region of chemical space. This compactness is consistent with the previously observed reduction in chemical diversity for (RA + M1), as reflected by increased Tanimoto similarities among the strongest binders.

The RA-SYNS remained closer to its starting chemical space throughout GAL and did not effectively traverse new regions of chemical space. This limited exploration is consistent with earlier observations that RA failed to identify strong binders and produced comparatively weaker binders.

In contrast, both M1 and CAZP rapidly moved away from the original chemical space after the first GAL iteration. Following this initial shift, only limited further movement in chemical space was observed in subsequent iterations. This behavior is consistent with earlier results showing that the strongest binders for M1 and CAZP were predominantly identified after the first iteration, with limited improvement in binding affinity in later iterations.

The novelty of generated strongest binders for TNKS2 was evaluated by comparison with previously published binding compounds. Binders were collected through the ChEMBL database, selecting compounds with IC_50_ < 1 μM, resulting in 604 binding compounds. Compounds were compared using the Tanimoto similarity of the Morgan fingerprints of their Murcko scaffolds. The results are shown in Table 1. When using SYNS, only in the case of RA + M1 a significant number of binders with similarity to known binders were observed and to a lesser extent for CAZP. Overall, however, the number of compounds with new scaffolds comprises the majority by far and indicates that identified binders are mostly genuinely novel.

**Table 1:** Ratio of 25 Generated Strongest Binders with Scaffolds Similar to Previously Published Binders for GAL with Different Synthesizability Scores

Tanimoto^a^ Similarity	No SYNS	M1	RA	RA + M1	CAZP
>0.5	0.56	0	0.04	0.36	0.24
>0.6	0.2	0	0	0.12	0
>0.7	0.2	0	0	0	0

a Tanimoto similarities of Morgan fingerprints of Murcko scaffold pairs were measured.

Overall, these results demonstrate that, except for RA, the synthesizability scoring schemes not only enable the discovery of compounds with high binding affinity and high synthesizability but also drive exploration toward novel regions of chemical space that are well separated from the structures used to initialize GAL.

#### 3.2.7 Application of Synthesizability Scoring at Final GAL Step Only

To investigate the effect of applying synthesizability scoring only at the final stage of GAL, we analyzed the binding affinity and chemical diversity of the 25 strongest binding compounds when SYNS were introduced exclusively in the last iteration, effectively as a post-processing step. Figure 8 shows the resulting distributions of binding free energies (Figure 8a) and pairwise Tanimoto similarities (Figure 8b) of the 25 best binders after each iteration, for TNKS2.

**Figure 8:**
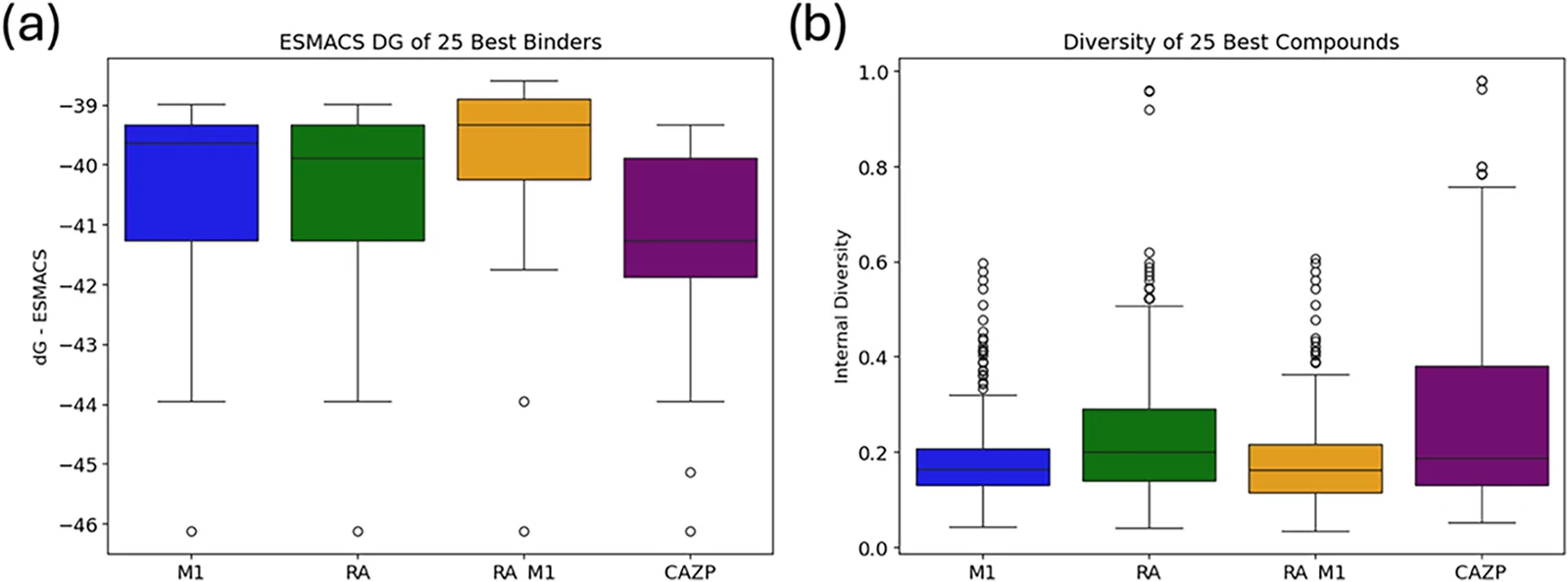
Distributions of binding free energies (a) and Tanimoto similarities of molecule pairs (b) of molecules taken from the 25 strongest binding compounds of accumulatively generated compounds, where M1, RA, RA + M1, and CAZP SYNS were only used at the last step of GAL.

Despite the absence of synthesizability constraints during the early stages of GAL, strong binding affinities were still obtained after applying synthesizability filtering at the final step. Median binding free energies of approximately −39 to −40 kcal/mol were observed for the M1, RA, and RA + M1 scoring schemes, while CAZP achieved a median binding free energy of −41 kcal/mol. For CAZP and M1, these results are comparable to those obtained when synthesizability scoring was applied from the beginning of GAL. In contrast, the performance of RA and RA + M1 improved substantially.

Also, when synthesizability scoring was applied only at the final iteration, a high degree of chemical diversity was retained among the strongest binders. The median Tanimoto similarity across the top 25 compounds was approximately 0.2, indicating substantial structural diversity in this compound ensemble. This diversity is notably higher than that observed when SYNS were applied throughout the entire GAL process, suggesting that early unconstrained exploration enables broader sampling of chemical space.

These results suggest that, for the TNKS2 target, synthesizability constraints do not necessarily need to be applied during the entire GAL process to obtain strong and synthetically favorable binders. Instead, applying synthesizability scoring as a final selection criterion may be sufficient, allowing GAL to fully exploit chemical space during early iterations while still producing practically viable candidates.

In the following section, we extend this analysis to the 3CL^pro^ target to assess whether this conclusion remains valid for a more challenging target characterized by variations in ligand binding modes as observed in our preceding work.

### 3.3 GAL Performance for Test Case 3CL^pro^

#### 3.3.1 Performance of GAL-Surrogate Model

As before, the quality of the surrogate model for the 3CL^pro^ target was evaluated during GAL without SYNS and with SYNS, by comparing Δ*G*_bind_ of the surrogate model with ESMACS predictions. Surrogate performances are shown in Figures S9–S10 and for CAZP, the surrogate quality is compared with GAL when no SYNS was used in Figure 9.

**Figure 9:**
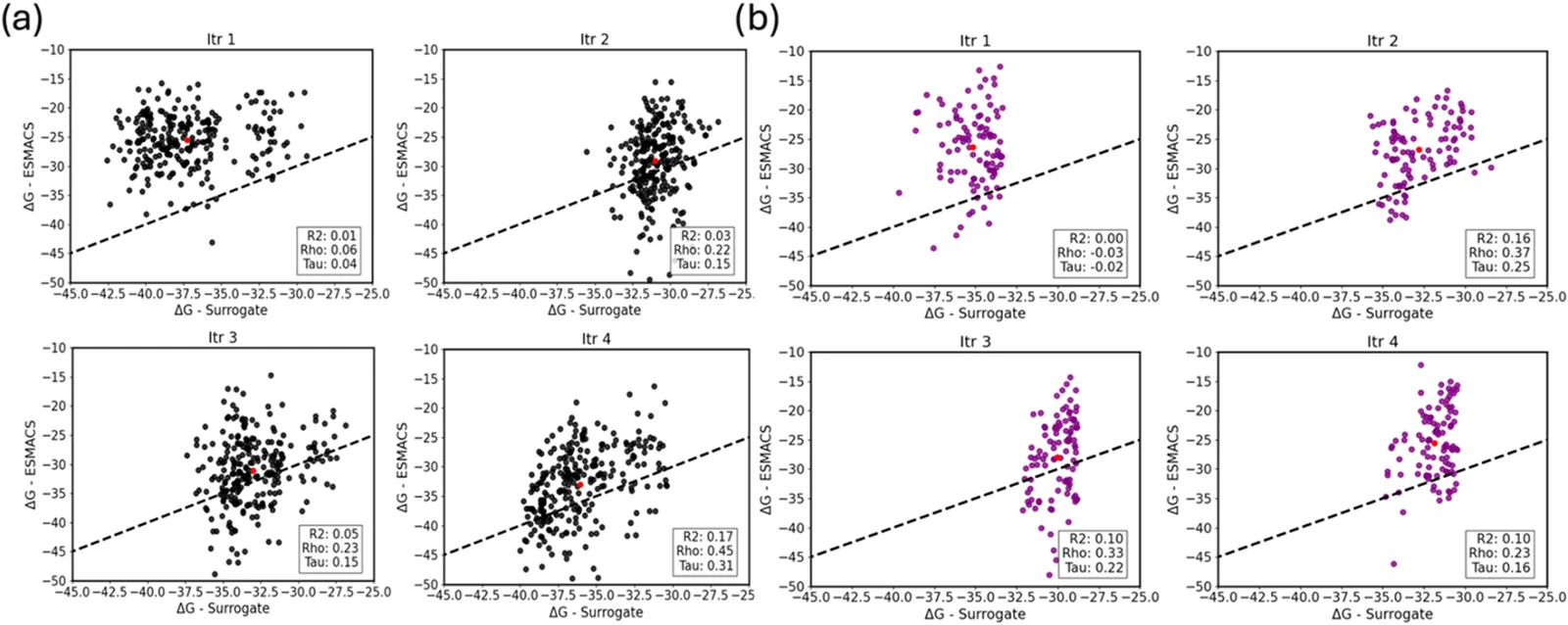
Comparison of Δ*G* predicted by the surrogate model with predictions from the ESMACS oracle for different iteration steps of GAL for 3CL^pro^ when no SYNS were used (a) and with CAZP (b). The *R* ^2^ coefficient, Spearman’s ρ, and Kendall’s τ are given for individual iterations and are also given in the insets. Average values of Δ*G* from surrogate and ESMACS are indicated with a red dot. All energies are given in kcal/mol.

Compared to TNKS2, the surrogate model generally exhibited weaker predictive performance for 3CL^pro^, consistent with observations from our previous work. In the absence of synthesizability scoring, the surrogate model achieved a maximum Spearman ρ = 0.45 by iteration four (Figure 9a), indicating limited but non-negligible ranking power.

When SYNS were introduced, surrogate model performance deteriorates further. Among the different scoring schemes, only CAZP achieved a Spearman correlation of ρ = 0.37 and 0.33 at iteration steps two and three, respectively (Figure 9b). For all other synthesizability scoring schemes, the surrogate predictions showed little to no correlation with the ESMACS oracle results (Figure S10). Under these conditions, compound selection for oracle evaluation was effectively close to a random selection, with only a small number of true positives that were forwarded.

Despite this weak surrogate performance, it is important to note that GAL does not require highly accurate surrogate models to be effective. As demonstrated in our previous work,^33^ even a limited number of generated true positives can provide sufficient signal for GAL to improve binding properties over successive iterations. Overall, the results for 3CL^pro^ highlight the increased difficulty to optimize compounds for this target compared to TNKS2.

#### 3.3.2 Binding Affinity Distributions of Generated Compounds

In the next step Δ*G*_bind_–distributions of generated molecules were analyzed in Figure 10, as predicted by the ESMACS oracle, both without synthesizability scoring and with different synthesizability scoring schemes applied as before.

**Figure 10:**
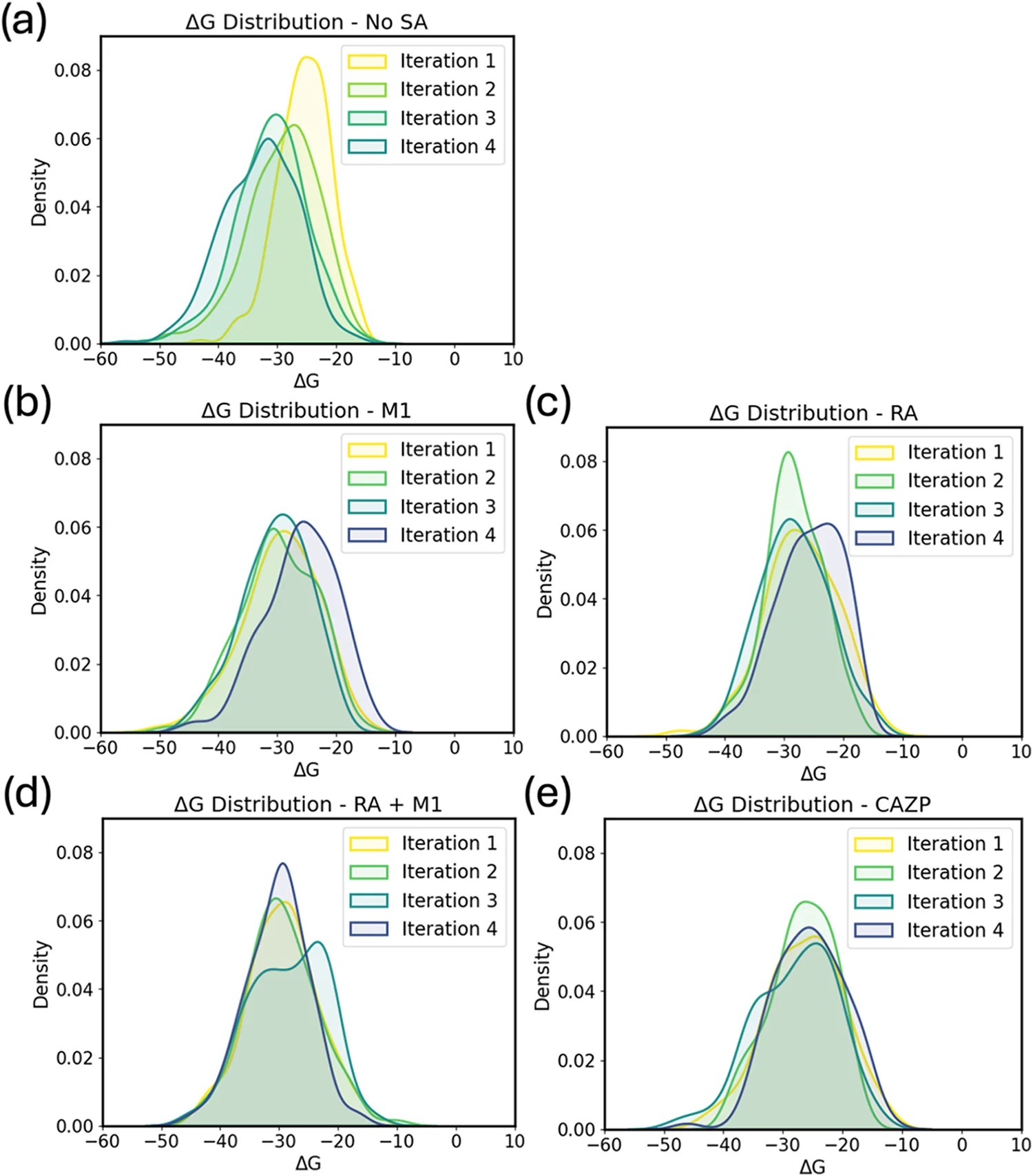
Distribution of Δ*G*_bind_ of compounds evaluated with ESMACS after each GAL iteration for 3CL^pro^, without SYNS (a) and using synthesizability scoring with M1 (b), RA (c), RA + M1 (d), and CAZP (e) methods.

In the absence of synthesizability scoring, a steady improvement in the Δ*G*_bind_ distributions is observed over successive GAL iterations. This improvement is reflected by a progressive shift of the distribution maximum toward lower Δ*G*_bind_ values, corresponding to stronger binding affinities. This indicates that GAL was able to consistently identify increasingly favorable binders for 3CL^pro^ when unconstrained by synthesizability considerations.

In contrast, when SYNS were applied, the Δ*G*_bind_ distributions showed little improvement after the first GAL iteration and, in some cases, even shifted toward weaker binding affinities in subsequent iterations. This behavior suggests that the inclusion of synthesizability constraints substantially restricts the ability of GAL to identify improved binders in the case of 3CL^pro^.

The notable exception was the CAZP score (Figure 10e). When CAZP was used, a more pronounced low-Δ*G*_bind_ tail developed during GAL, indicating enrichment with stronger binders. This observation is consistent with results shown above that CAZP is the only synthesizability scoring scheme for which the surrogate model exhibited measurable correlation with ESMACS oracle predictions, albeit limited. As a result, more true positives were identified and forwarded to the oracle that provided sufficient signal for GAL to improve Δ*G*_bind_.

Overall, these results highlight the sensitivity of GAL performance for 3CL^pro^ to the quality of the surrogate model and the choice of synthesizability scoring scheme and underscore the greater difficulty of this target compared to TNKS2.

#### 3.3.3 Distribution of Synthesizability Scores of Generated Compounds

Figure S11 shows the distributions of SYNS for 3CL^pro^ compounds evaluated with the ESMACS oracle after each GAL iteration, using different scoring methods.

For M1, RA, and CAZP, the majority of compounds achieved SYNS in the range of 0.9–1.0, indicating that GAL is able to efficiently identify compounds that satisfy the compound synthesizability objective. The combined RA + M1 scoring scheme exhibits a broader score distribution, similar to the situation for TNKS2 but also in that case most scores are in the range 0.9–1.0.

Overall, these results demonstrate that the secondary optimization objective of identifying synthetically favorable compounds was successfully achieved for 3CL^pro^. However, as shown in the preceding sections, satisfying this objective does not necessarily translate into improved Δ*G*_bind_-values for this target.

#### 3.3.4 Chemical Diversity of the Strongest Binding Compounds

Chemical diversity of the compounds generated is shown in Figure 11, displayed as pairwise Tanimoto similarity distributions derived from Morgan fingerprints of the compounds, for the cumulatively best 25 compounds evaluated with ESMACS for 3CL^pro^ as before.

**Figure 11:**
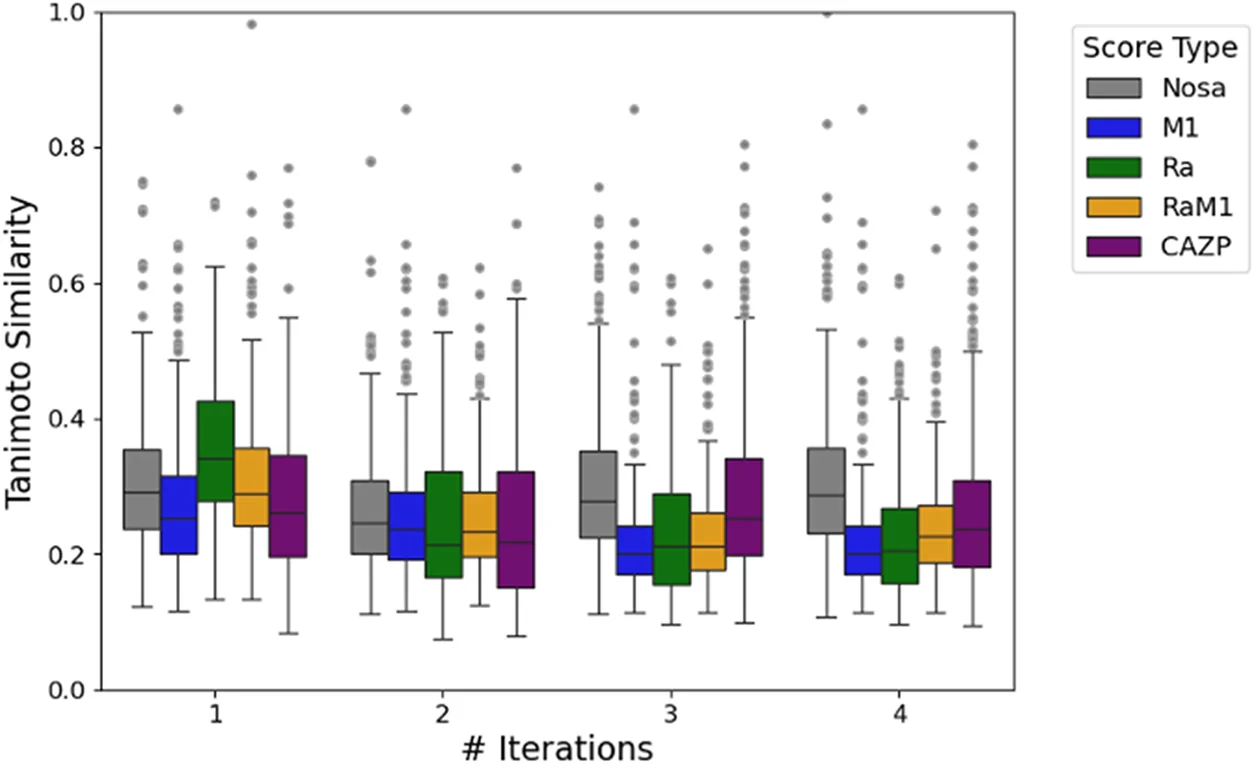
Tanimoto similarity distributions of molecule pairs, taken from the 25 strongest binding compounds of accumulatively generated compounds evaluated with ESMACS and evaluated after each GAL iteration for 3CL^pro^, with M1, RA, RA + M1, and CAZP synthesizability scoring and without SYNS.

In the absence of synthesizability scoring, the median Tanimoto similarity is approximately 0.3, indicating molecular diversity within the strongest binding compounds.

After four GAL iterations with SYNS applied, median Tanimoto similarities decreased to values around 0.2, indicating increased chemical diversity relative to the unconstrained case. This level of diversity was also higher than that observed for TNKS2 under comparable conditions. The only exception is the RA-SYNS, which exhibited similarly high chemical diversity for both TNKS2 and 3CL^pro^.

Overall, these results indicate that for 3CL^pro^, the introduction of synthesizability constraints does not introduce more structural redundancy among the strongest binders in the compound ensemble.

#### 3.3.5 Binding Affinity Distributions of the Strongest Binding Compounds

Δ*G*_bind_-distributions of the 25 strongest binding compounds identified cumulatively across GAL iterations for 3CL^pro^ are shown in Figure 12. Identifying new compounds with strong binding affinity for the target was the main objective of GAL. An average standard error of Δ*G*_bind_ = 1.8 kcal/mol was derived from affinity variation across 20 simulation replicas. This error is larger than in the case of TNKS (0.8 kcal/mol) as expected due to the larger ligand flexibility in the more open 3CL^pro^ binding pocket.

**Figure 12:**
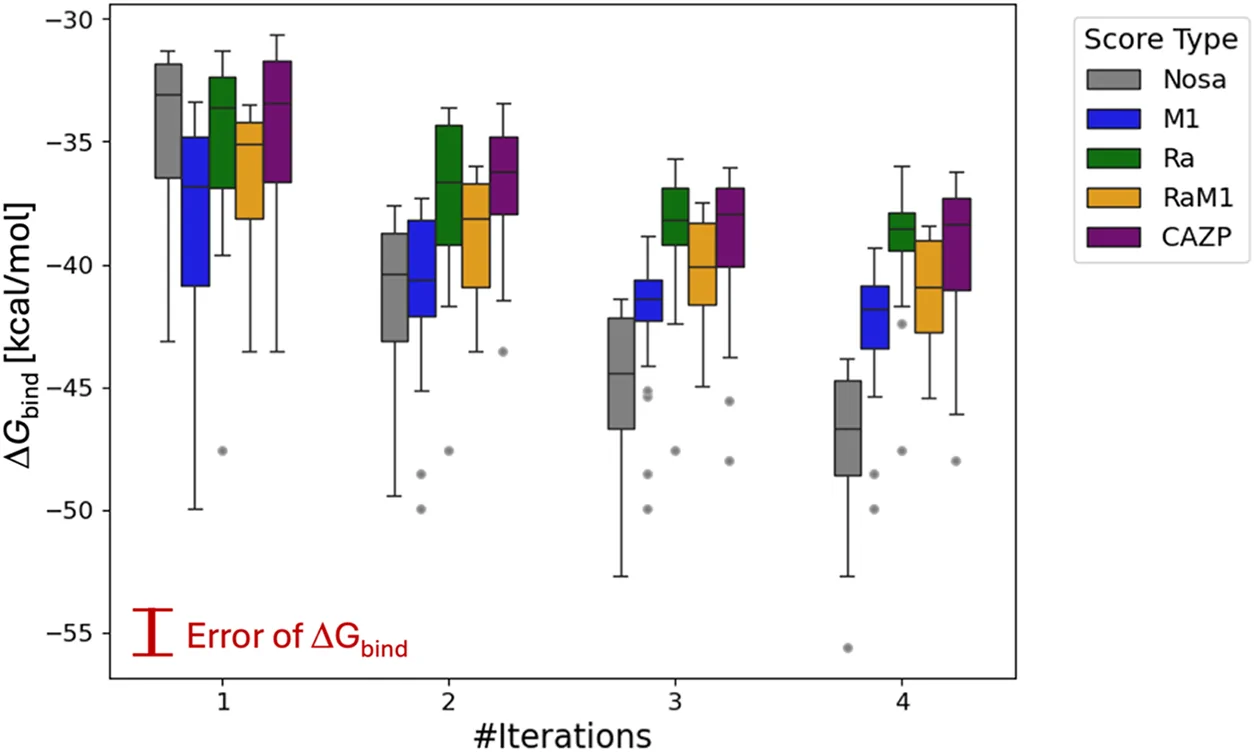
Binding free energy distribution of molecules taken from the 25 strongest binding compounds of accumulatively generated compounds evaluated with ESMACS and evaluated after each GAL iteration for 3CL^pro^, with M1, RA, RA + M1, and CAZP synthesizability scoring and without SYNS. The standard error of Δ*G*_bind_ = 1.8 kcal/mol is indicated for comparison in the bottom left corner.

In the absence of SYNS, a steady improvement in the average binding free energy was achieved throughout GAL. After four iterations, median Δ*G*_bind_ values reached approximately −47 kcal/mol, indicating successful identification of increasingly strong binders after each iteration.

When SYNS were added, improvements in the median Δ*G*_bind_ values from one iteration to the next were still achieved across all four scoring schemes, even though the overall Δ*G*_bind_-distributions of all evaluated compounds changed only marginally, as discussed previously, with the exception of scoring with CAZP. This indicates that GAL was still able to enrich the pool of strongest binders under synthesizability constraints. Also, as shown in Figure 12, the five best binders found with CAZP–scoring achieved values of Δ*G*_bind_ comparable to those found with M1.

Nevertheless, in all cases the strongest binders identified with synthesizability scoring exhibited weaker binding affinities compared to the unconstrained GAL setup. Among the four scoring schemes, M1 performed best, reaching a median Δ*G*_bind_ of −42 kcal/mol, although it also showed the smallest improvement relative to iteration one. The combined (RA + M1) score achieved a median Δ*G*_bind_ values of −41 kcal/mol after the last iteration, i.e., comparable to the value with M1 scoring alone, while RA and CAZP both only reached −38 kcal/mol, which is significantly weaker. The CAZP scoring scheme would likely have benefited from additional GAL iterations, as the overall Δ*G*_bind_ distribution continued to improve up to the final iteration.

Overall, in contrast to TNKS2, the inclusion of synthesizability scoring as a second optimization objective for 3CL^pro^ led to a noticeable reduction in the binding affinity of the strongest binders, on the order of 5 kcal/mol or more. Despite this reduction, GAL successfully identified compounds with high SYNS and substantial chemical diversity among the strongest binders, highlighting a clear trade-off between binding affinity and synthesizability for this more challenging target.

In the next step, the chemical structures of the strongest binders were examined generated with GAL using different SYNS. Figures S12–S16 show the 25 strongest binding compounds identified at the end of GAL without SYNC and with M1, RA, M1 + RA, and CAZP. The 5 strongest binders without SYNC and with CAZP and M1 are shown in Figure 13. The compounds are depicted in order of decreasing binding affinity as estimated by ESMACS.

**Figure 13:**
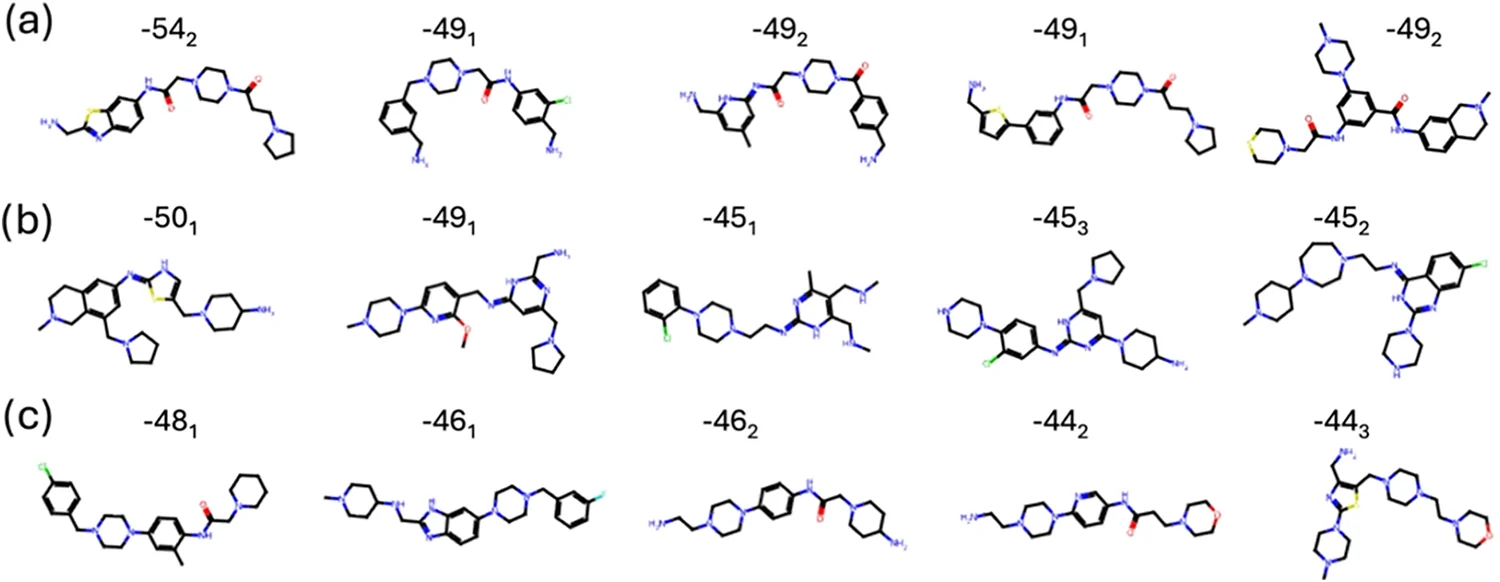
Five strongest binding compounds, depicted in order of decreasing affinity strength according to ESMACS for 3CL^pro^, found without SYNS (a), with M1 (b) and CAZP (c) at the end of GAL. Numbers are binding free energies of compounds and are given in kcal/mol together with standard errors as index. All 25 best binding compounds are shown in Figures S12–S16.

Diversity of scaffolds was investigated using Murcko scaffolds as described above. For M1 and RA all scaffolds were unique, for RA + M1 only one duplicate scaffold was found and for CAZP one scaffold was found three times in the ensemble. Average Tanimoto similarities of Murcko scaffolds were found to be <0.3 in all cases. As it was the case for TNKS2, also for 3CL^pro^ scaffolds often share common motives and could be assigned the same scaffold family. Scaffold similarity was found to be generally lower than for TNKS2, which could be a result of the more open binding pocket that allows more structural variety of binders.

Notable differences in chemical structures are observed when comparing CAZP- and M1-generated compounds. This finding again indicates that GAL with different SYNS converges to different areas in chemical space.

In case of CAZP, the core consists of a linear and polar heterocyclic backbone with numerous nitrogen substitutions, typically containing amide linkage and a carbonyl group. Compounds generated with M1 tend to be more nonlinear with branching, showing more variations in the scaffold, and mostly composed of polar heterocyclic rings with nitrogen substitutions, similar to CAZP compounds, often linked through amide, carbonyl or alkyl groups.

Compounds generated with CAZP synthesizability scoring are predominantly linear in shape and show less variation in overall molecular geometry, which was also observed to a lesser extend for TNKS2. This reduced variability in structure suggests that the CAZP score favors such elongated and linear molecular architectures when compared to M1. However, chemical variation according to Tanimoto similarities showed that M1 and CAZP exhibit overall comparable diversity, according to results in Section 3.3.4

#### 3.3.6 Exploration of Chemical Space during GAL

We also examined chemical space traversal during the optimization process using t-SNE projections of Morgan fingerprints, shown in Figure 14, for the first two t-SNE components of the 25 strongest binders for 3CL^pro^.

**Figure 14:**
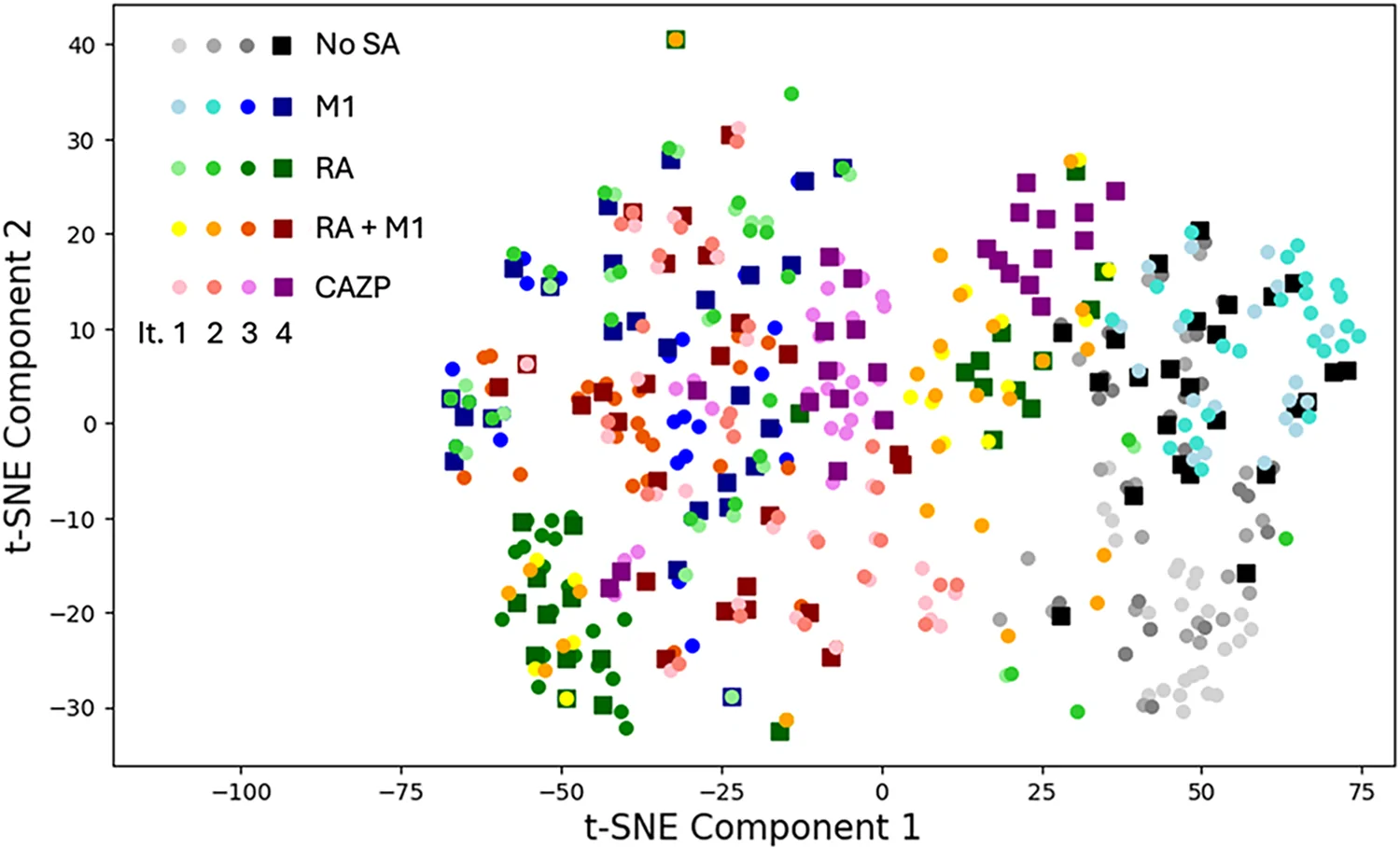
First two t-SNE components of Morgan fingerprints of the 25 strongest binding molecules to 3CL^pro^ of accumulatively generated compounds after each GAL iteration for 3CL^pro^, where synthesizability scoring has been used throughout GAL and without SYNS (No SA).

When no SYNSs were applied, a clear movement through chemical space is observed, with the best binders becoming increasingly distinct from the structures used to initialize GAL. This qualitative observation does not change when synthetic accessibility scores are added.

Notably, different SYNS led mostly to convergence into distinct regions of chemical space. After iteration four, compounds generated using M1 and M1 + RA are more or less evenly dispersed across a relatively large area of chemical space, with substantial overlap between their respective distributions. In contrast, the RA and CAZP distributions form tighter clusters, consisting of two and three well-separated regions, respectively, indicating more localized exploration.

Comparing iterations 3 and 4 reveals that the M1 and M1 + RA distributions change only marginally, suggesting that these runs had largely converged. In contrast, the RA and CAZP distributions continue to shift noticeably between these iterations, indicating that convergence had not yet been fully achieved. This observation is consistent with the previously discussed behavior of the Δ*G*_bind_ distributions in Section 3.3.2, which also continued to shift for RA and CAZP at later iterations.

Also, for 3CL^pro^ the novelty of the strongest binders generated was evaluated by comparison with previously published binding compounds with the method described in Section 3.2.6. 1749 binders were found in ChEMBL. The results are shown in Table 2. Compared to TNKS2, the dissimilarity of generated binders and known binders is even larger, and essentially all identified binders exhibit scaffolds well distinct from what has been previously published. Together with the results for TNKS2, these findings demonstrate that the GAL approach is indeed capable of identifying binders in new chemical spaces.

**Table 2:** Ratio of 25 Generated Strongest Binders with Scaffolds Similar to Previously Published Binders for GAL with Different Synthesizability Scores

Tanimoto^a^ Similarity	No SYNS	M1	RA	RA + M1	CAZP
>0.5	0.08	0	0	0	0.04
>0.6	0.04	0	0	0	0
>0.7	0	0	0	0	0

a Measured were Tanimoto similarities of Morgan fingerprints of Murcko scaffold pairs.

Overall, we observed that inclusion of SYNS in the GAL optimization for the more demanding target 3CL^pro^ led to a reduction in binding affinity of the 25 best binders in contrast to the case of TNKS2. Application of M1 scores resulted in the strongest binding affinities; however, when looking at the ten top binders, results from CAZP were found to be comparable. The second optimization goal, high SYNS of generated compounds, was achieved in all cases. Chemically and structurally diverse compounds, well distinct from the molecules used to initialize GAL, were found with all synthesizability scoring schemes. Results could probably be improved by using a more predictive surrogate model to enable better selection of compounds for the ESMACS oracle evaluation. The quality of surrogate model predictions was limited and only preserved to some extent with CAZP scores, probably due to the varying binding modes of ligands in 3CL^pro^ in a more open and larger binding pocket, as already observed in our previous work.

#### 3.3.7 Application of Synthesizability Scoring at Final GAL Step Only

For this target, we also investigated the effect of applying synthesizability scoring only at the final iteration of GAL and compared the results to the previous case where synthesizability scores were used for all GAL steps. Figure 15 shows the resulting distributions of binding free energies of the 25 best binders for the 3CL^pro^ target.

**Figure 15:**
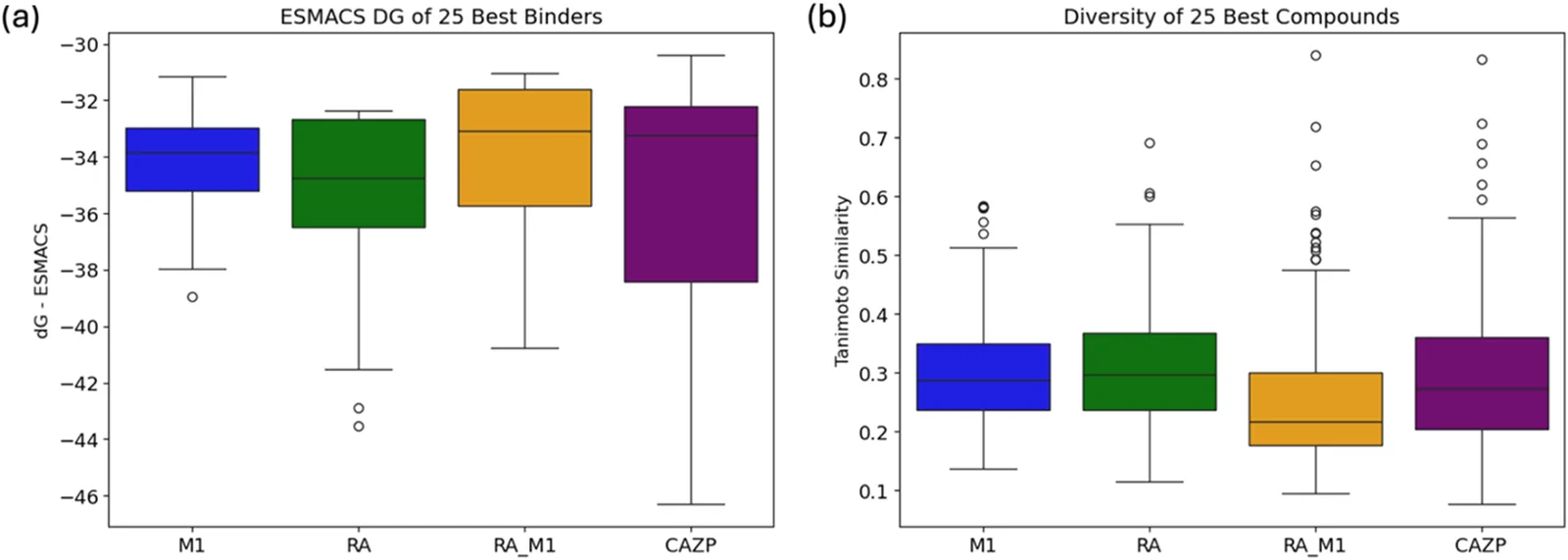
Distributions of binding free energies (a) and Tanimoto similarities of molecule pairs (b) of molecules taken from the 25 strongest binding compounds of accumulatively generated compounds, where M1, RA, RA + M1 and CAZP–SYNS were only used at the last step of GAL for 3CL^pro^.

When focusing exclusively on the best binders, the average predicted Δ*G*_bind_-values were relatively similar across all four SYNS. RA achieved a median Δ*G*_bind_ of −35 kcal/mol, followed by M1 at −34 kcal/mol, while CAZP and M1 + RA both yielded medians of −33 kcal/mol. Despite these insignificantly different median values, the CAZP distribution extended further into lower Δ*G*_bind_ values, indicating a greater ability to identify the strongest individual binders.

Compared to runs where SYNS were applied from the start, the ability to identify strong binders declined substantially for M1 and M1 + RA when synthesis constraints were introduced only in the final iteration. A similar but less pronounced decline was also observed for RA and CAZP scores.

Analysis of molecular diversity showed average Tanimoto similarities in the range of 0.26–0.30 for all methods except M1 + RA, which exhibited lower similarity values around 0.2. Relative to runs with early application of SYNS, diversity decreased for the M1 and RA methods, while remaining largely unchanged for M1 + RA and CAZP.

Overall, for the more challenging 3CL^pro^ target, these results indicate that applying SYNS only at the final stage of GAL leads to inferior performance compared to incorporating them from the outset. Early inclusion of synthesis constraints appears necessary to effectively guide GAL toward optimal binding affinity while maintaining favorable SYNS.

## 4 Discussion

A central question addressed in this work is whether physics-guided GAL, as presented in our previous work for binding affinity optimization, can be extended to a more realistic multi-parameter optimization problem without substantial performance loss. In drug discovery, binding affinity alone is insufficient, since compounds must also be synthetically accessible. The introduction of synthesizability as a second objective therefore represents a critical step toward practical application in drug discovery projects.

One encouraging observation was that synthesizability constraints did not universally lead to compounds with reduced predicted binding affinity. In fact, for TNKS2, M1, and CAZP preserved high-affinity tails comparable to the unconstrained setup. Only the use of RA alone substantially limited optimization. This suggests that some synthesizability scores effectively filtered out problematic compounds without severely restricting traversal of chemical space during the GAL. In contrast, for 3CL^pro^, all scoring schemes led to reduced affinity gains. This likely reflects a more complex structure–affinity relationship that led to a weaker surrogate model performance. In that case, acquisition of compounds to the ESMACS oracle occurs to some extent randomly. Under such conditions, adding synthesizability constraints burden exploration to a degree that limits the discovery of high-affinity compounds by adding more dimensions to the optimization problem. Nevertheless, improvements in binding affinity throughout GAL were still achieved also in this challenging case.

Another practical finding was that applying synthesizability only in the final GAL iteration preserved both high binding affinity and chemical diversity for TNKS2. This suggests a two-phase strategy combining unconstrained exploration followed by constrained exploitation can be a viable procedure for some protein targets, when the structure–affinity relationship is sufficiently straightforward and affinity surrogate models with ranking power can be established. The two-phase approach is also interesting as it reduces computational expense, which in case of CAZP is significant. However, when more complex binding sites are involved as it is the case for 3CL^pro^, a surrogate model with limited performance could already limit effective exploration of chemical space before the post-processing filtering. The t-SNE projections and scaffold inspection demonstrate that different synthesizability scores bias GAL toward distinct chemical regions. This implies that synthesizability scores are not only neutral filters that remove problematic compounds but that they could also actively influence the direction of the generative process, i.e., synthesis score biases could accordingly translate into biases of the compounds generated.

Several limitations of the presented approach are acknowledged. The binders identified in the present study have not been experimentally confirmed, which was beyond the scope of the present work. In related work, we applied the same physics-guided GAL workflow; however, using an FEP-based oracle, where results were indeed validated experimentally for a different target, WDR91.^88^ This provides complementary evidence that the overall GAL approach is suitable to translate computational optimization into experimentally confirmed binders, while experimental validation for the specific GAL used here, remains for future work.

Moreover, synthesizability scores are approximations, and high scores do not guarantee successful synthesis as this, apart from inaccuracies of the model, also depends on the experience and resources of the synthesis lab. Synthesis scoring schemes are also biased to some extent towards reactions and scaffolds used for training, thereby causing inaccuracies when applied to deviating chemistry. Also, reactions reported as feasible and used for training may not be reproducible as important experimental parameters are often missing in the dataset. Furthermore, mapping the complexities involved in a series of several reaction steps to a single score is inevitably limited in expressiveness to describe the difficulty to synthesize a compound in practice. Therefore, a diverse set of high-affinity binders with high synthesis scores would still need to be shortlisted by experienced synthesis chemists. This is the reason why high chemical diversity of GAL generated compounds is crucial. Importantly, our results indicate that, indeed, the inclusion of synthesis constraints does not seem to compromise the generation of a chemically and physically diverse set of compounds with GAL.

Limitations in the accuracy of ESMACS compared to alchemical free energy approaches are well compensated by enabling high throughput simulations as necessary for hit finding. Also, ESMACS enables derivation of absolute binding affinities that are challenging to compute using alchemical approaches which are better suited for relative binding affinity calculations as encountered in lead optimization. Lastly, a further limitation is that GAL, with the underlying MD simulations involved in ESMACS and the reinforcement learning of Reinvent, are of stochastic nature and non-deterministic, i.e., repetition of GAL could lead to convergence into different parts of chemical space. This limitation is mitigated to some extent by using the ESMACS ensemble approach, where ten replicas were used for each molecule, for a more robust derivation of binding affinities.

We expect future refinements of the approach presented here with the development of an improved surrogate model with enhanced binding affinity ranking power and inclusion of uncertainty-aware oracle acquisition. Also, phasing-in synthesizability scores more gradually rather than fully from the beginning or only at the last step could support traversal through chemical space toward regions of compounds with high binding affinity, while synthetically accessible. Finally, extending the presented GAL framework toward multi-parameter optimization including further scores to evaluate properties such as toxicity, ADME and solubility for instance, while maintaining exploration efficiency, remains an important challenge.

## 5 Conclusion

In this study, we demonstrated that physics-guided generative active learning can be extended from single-objective binding affinity optimization to a multi-objective framework that incorporates synthesizability constraints without fundamentally compromising performance. By combining generative modeling, surrogate learning, and ESMACS-based binding free energy estimation, we established a workflow capable of navigating chemically diverse search spaces under realistic design constraints. This approach is capable of identifying compounds that are not only strong binders but are simultaneously also synthetically accessible, presenting an important step toward practical drug design.

The impact of synthesizability constraints on affinity optimization is target-dependent and influenced by the interplay between binding site topology, surrogate ranking power, and the inductive biases introduced by synthesis scoring. While well-defined systems such as TNKS2 retained strong optimization performance under most scoring schemes, more complex systems such as 3CL^pro^ revealed trade-offs between affinity and synthesizability.

Overall, this work supports the feasibility of integrating physics-based binding free energy calculations with generative AI and synthetic accessibility scoring within a generative active learning approach. While challenges remain, particularly in maintaining ranking stability under increased objective complexity, the presented results provide encouraging evidence that multi-parameter molecular optimization can be pursued, while maintaining chemical space exploration efficiency.

## Supplementary Material



## Data Availability

The data underlying this study are available in the published article, the Supporting Information, and at 10.5281/zenodo.20442269. The code used to analyse the results is also available at 10.5281/zenodo.20442269.

## Notes

Performance of the GAL surrogate models for TNKS2 and 3CL^pro^ is shown for all iteration steps (Figures S1–S2 and S9–S10); distributions of achieved synthesizability scores are shown for TNKS2 and 3CL^pro^ for all iteration steps and different scoring schemes (Figures S3 and S11), and 25 best binding structures, according to ESMACS results, are shown for TNKS2 and 3CL^pro^, after the last GAL iteration step and for different synthesis scoring schemes (Figures S4–S8 and S12–S16) (PDF)
