# Supplementary material for: Generative Active Learning for Molecular Design with REINVENT: Balancing Binding Affinity and Synthetic Accessibility
Source: J Chem Theory Comput. 2026 Sep 8;22(18):9742–61. doi: 10.1021/acs.jctc.6c01017 (PMC13602925; doi:10.1021/acs.jctc.6c01017)
Supplement: Supplementary file 1 [file ct6c01017_si_001.pdf]

Supporting Information

Generative Active Learning for Molecular Design with REINVENT: Balancing Binding Affinity and Synthetic Accessibility

Marco Klähn[a *], Hannes H. Loeffler[a], Shunzhou Wan[b], Alexey Voronov[a], Xibei Zhang[b], Agastya P. Bhati[b,c *], and Peter V. Coveney[b,d *]

[a]Molecular AI, Discovery Sciences, R&D, AstraZeneca, Mölndal 431 83, Sweden
[b]Centre for Computational Science, Department of Chemistry, University College London, London WC1H 0AJ, U.K
[c]Wadhwani School of Data Science and Artificial Intelligence, Indian Institute of Technology Madras, Chennai, Tamil Nadu, India 600 036
[d]Advanced Research Computing Centre, University College London, London WC1H 0AJ, U.K.

This Supporting Information provides extended data, surrogate model evaluations and detailed structural analyses to complement the Generative Active Learning (GAL) framework described in the main manuscript. Performance benchmarks for the GAL surrogate models targeting TNKS2 and $3CL^{pro}$ across all iterative optimization cycles are presented in Figures S1–S2 and S9–S10, respectively. To evaluate synthetic tractability, Figures S3 and S11 detail the distributions of achieved synthesizability scores for both targets across all iterations and under varying scoring schemes. Finally, the structural outcomes of the workflow are compiled in Figures S4–S8 (TNKS2) and Figures S12–S16 ($3CL^{pro}$), which depict the top 25 highest-binding molecular conformations, as determined by ESMACS free-energy estimations, obtained after the final GAL iteration across the different synthesis scoring schemes.

In Figures S1-S2 the performance of the GAL surrogate model for TNKS2 is shown for all performed iteration steps to supplement Figure 2.

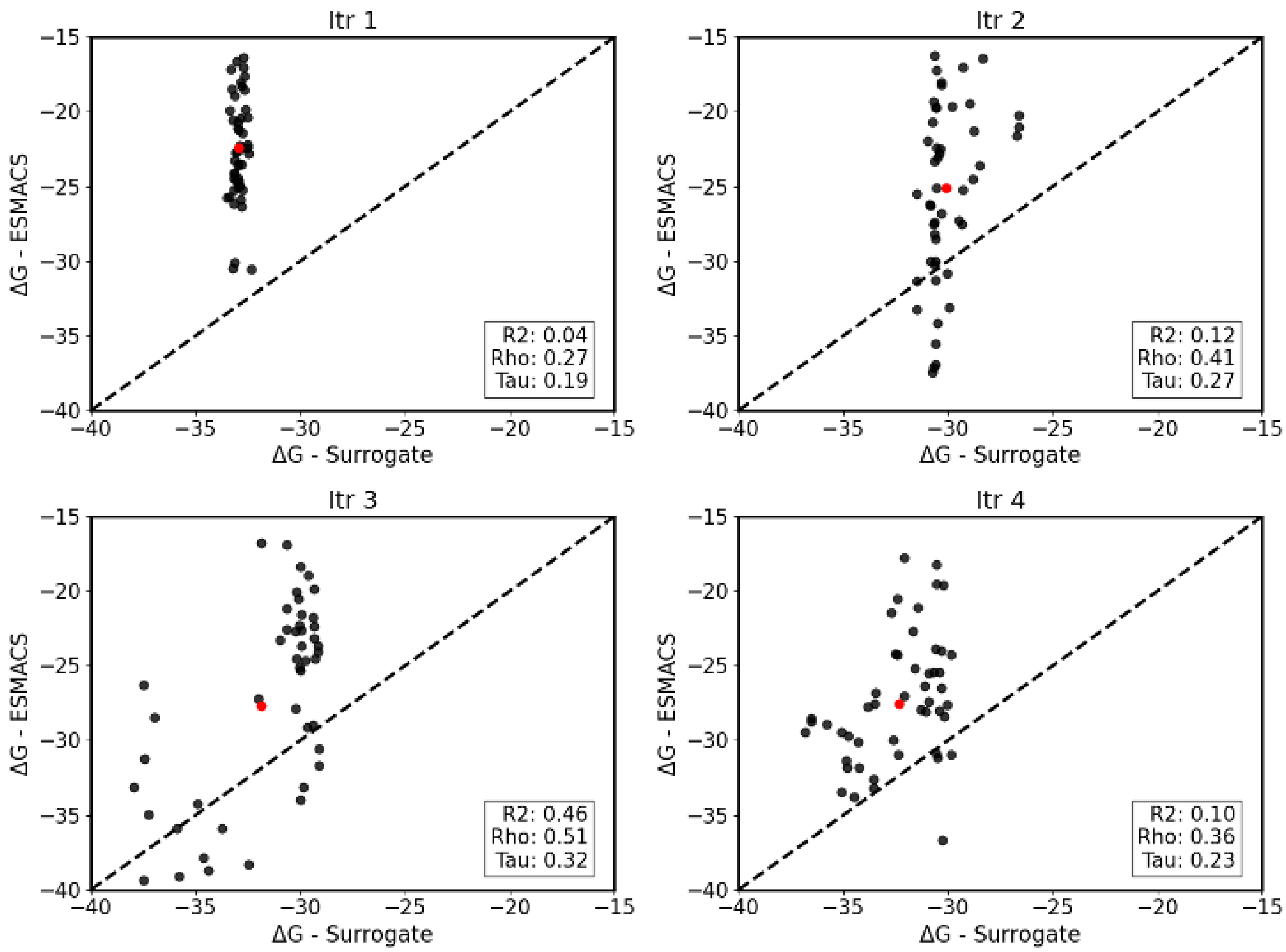

Fig S1: Comparison of ΔG predicted by the surrogate model with predictions from the ESMACS oracle for iteration steps 1 to 4 of the GAL for TNKS2. No SYNSs were used during GAL to provide a reference. The R^2 coefficient, Spearman's ρ, and Kendall's τ are given for individual iterations in the insets. Average values of ΔG from surrogate and ESMACS are indicated with a red dot. All energies are given in kcal/mol.

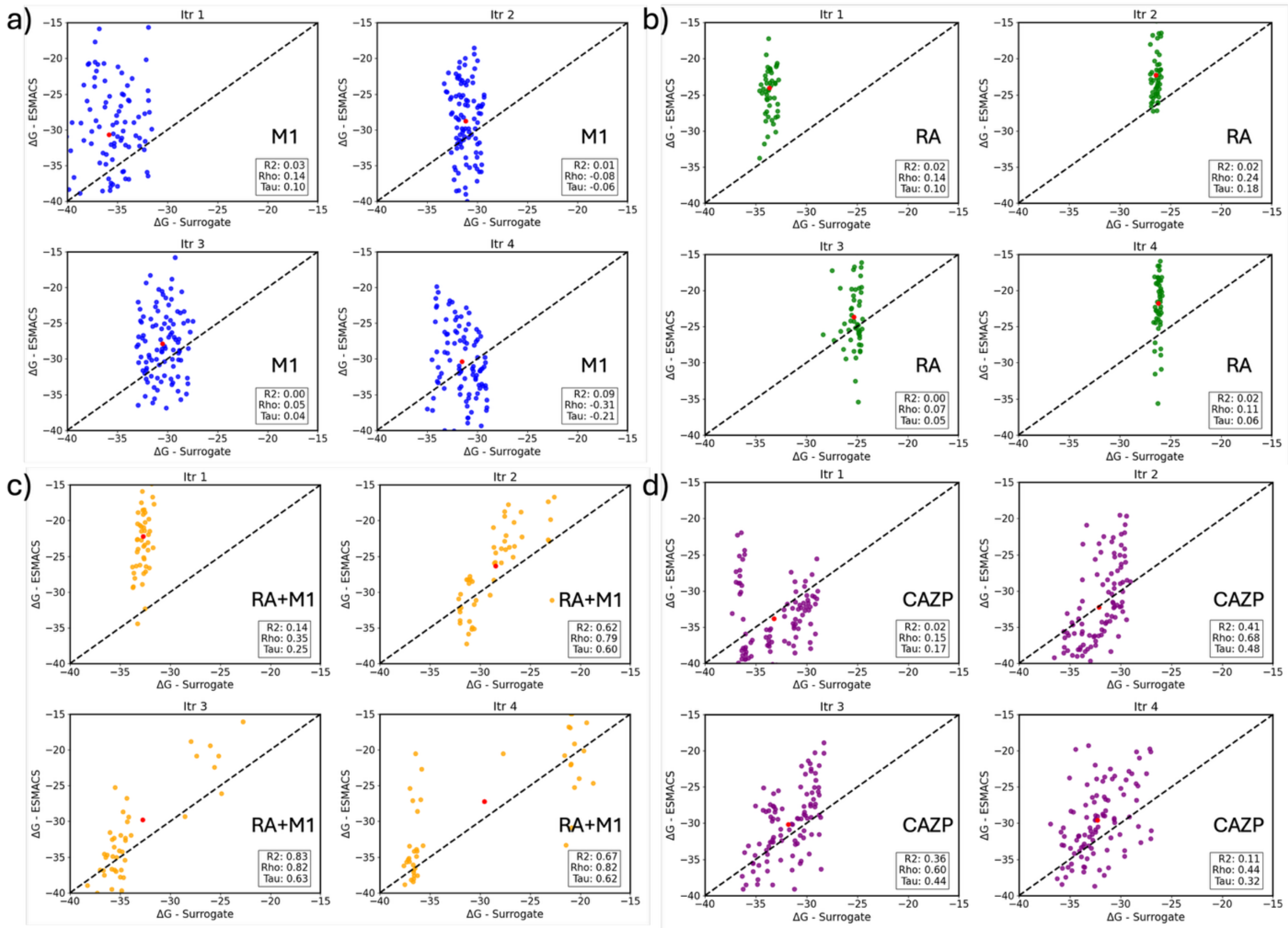

Fig S2: Comparison of ΔG predicted by the surrogate model with predictions from the ESMACS oracle for iteration steps 1 to 4 of the GAL for TNKS2. Synthesis scores M1 (a), RA (b), RA+M1 (c) and CAZP (d) were used during the AL. The R^2 coefficient, Spearman's ρ, and Kendall's τ are given for individual iterations in the insets. Average values of ΔG from surrogate and ESMACS are indicated with a red dot. All energies are given in kcal/mol.

In Figure S3, the distributions of achieved synthesizability scores are shown in case of TNKS2 for all iteration steps and different scoring schemes to support the discussion in section 3.2.3.

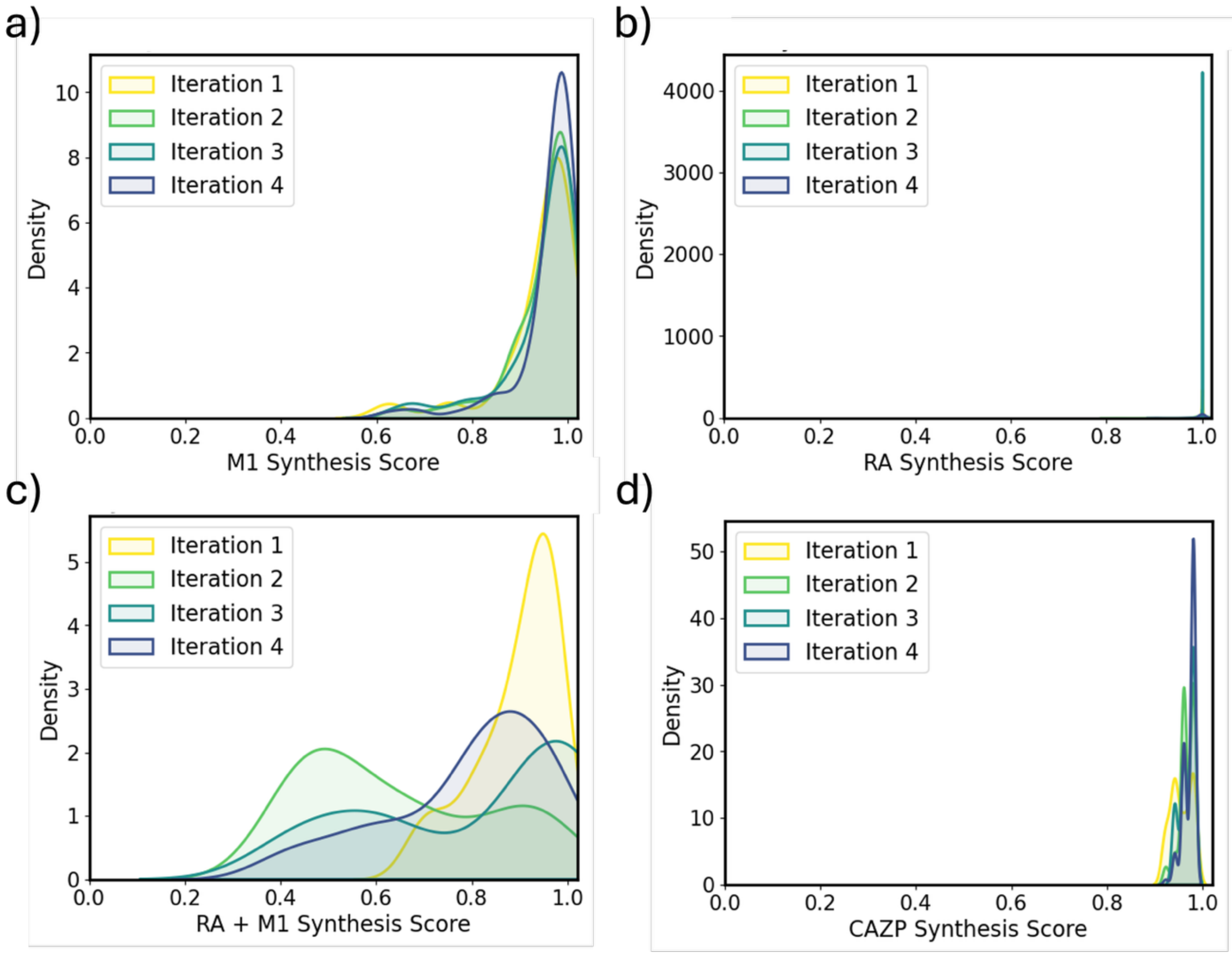

Fig S3: Distribution of synthesizability scores of compounds after each GAL iteration using scoring schemes M1 (a), RA (b), RA+M1 (c) and CAZP (d).

In Figures S4-S8, all 25 best binding structures, according to ESMACS results, are shown for TNKS2, after the last GAL iteration step and for different synthesis scoring schemes. These figures provide additional structures to Fig 6 in section 3.2.5 to demonstrate structural variety.

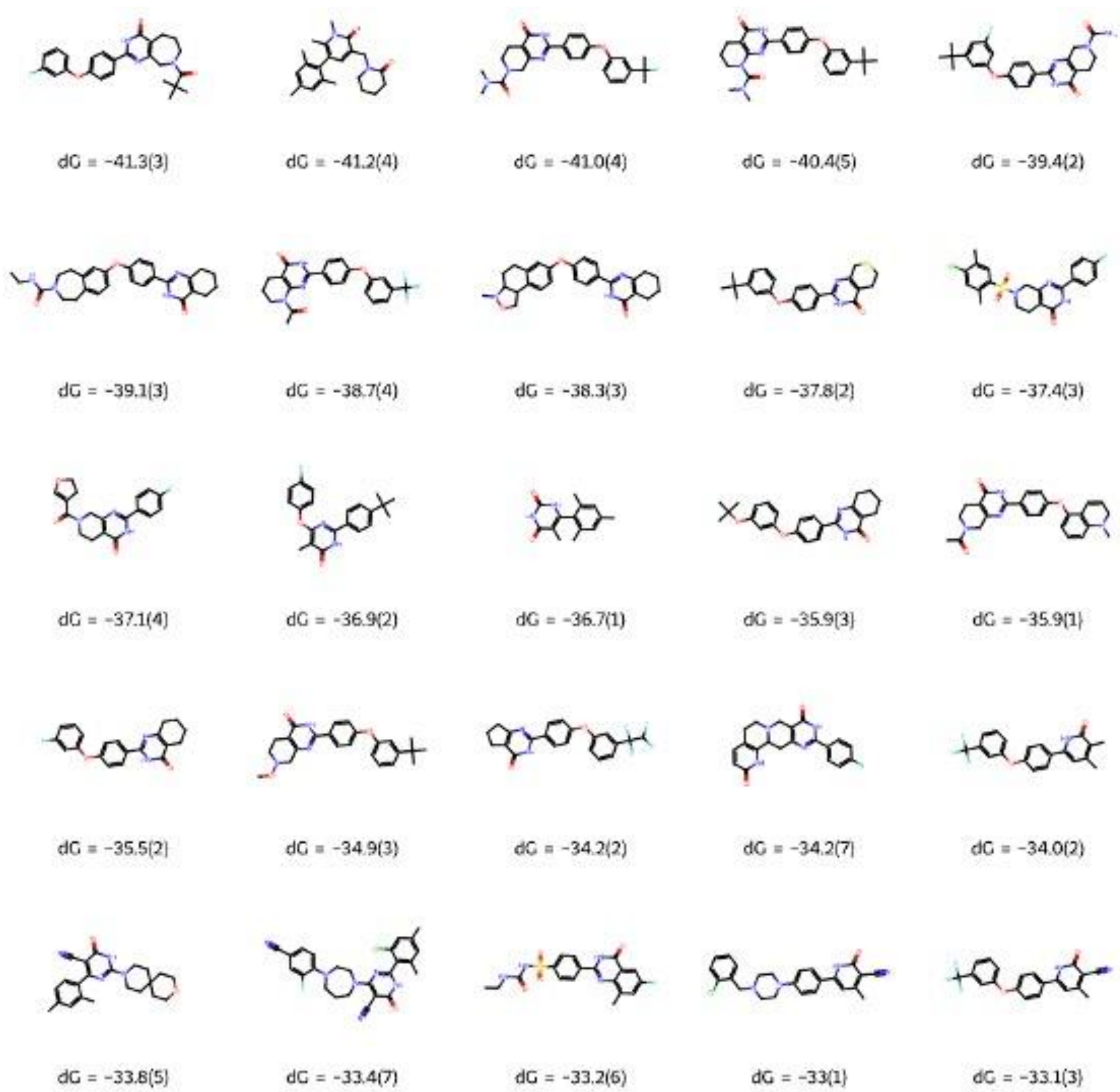

Fig S4: The 25 strongest binding compounds when no SYNS was used, depicted in order of decreasing binding affinity strength according to ESMACS for TNKS2, found with CAZP synthesis scoring at the end of GAL. Binding free energies are given in kcal/mol. Statistical errors are given in parenthesis.

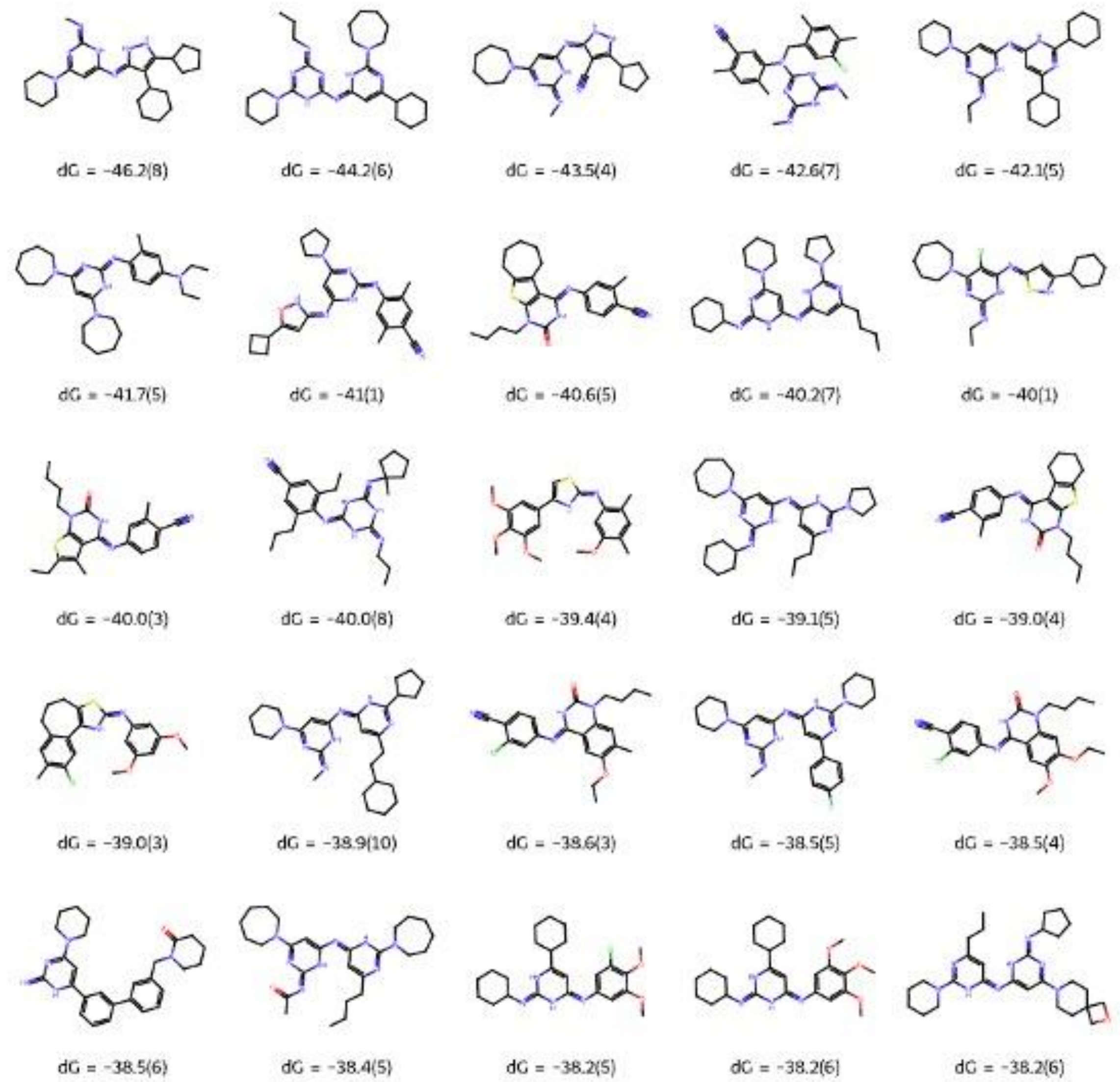

Fig S5: The 25 strongest binding compounds, depicted in order of decreasing binding affinity strength according to ESMACS for TNKS2, found with M1 synthesis scoring at the end of GAL. Binding free energies are given in kcal/mol. Statistical errors are given in parenthesis.

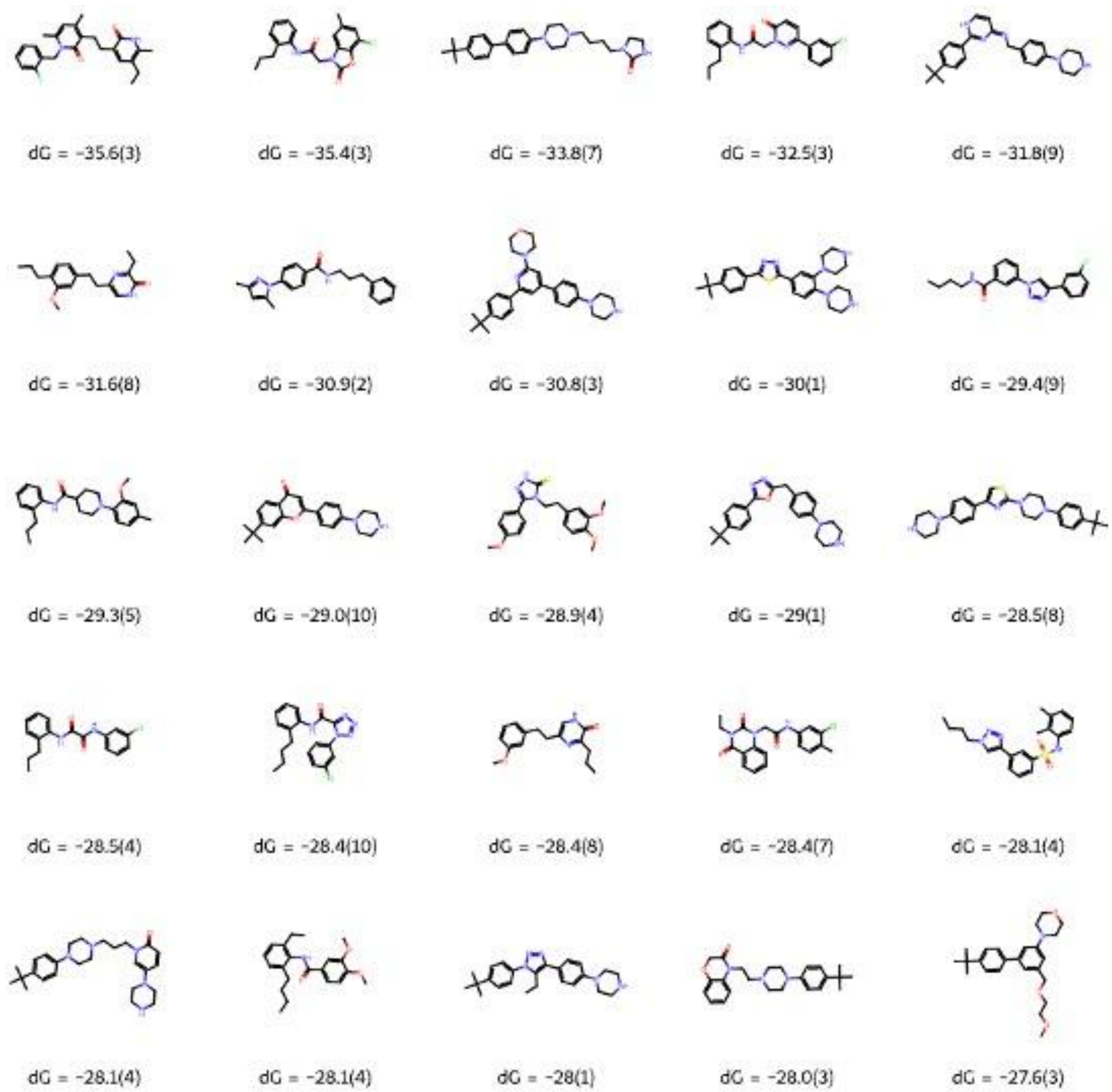

Fig S6: The 25 strongest binding compounds, depicted in order of decreasing binding affinity strength according to ESMACS for TNKS2, found with RA synthesis scoring at the end of GAL. Binding free energies are given in kcal/mol. Statistical errors are given in parenthesis.

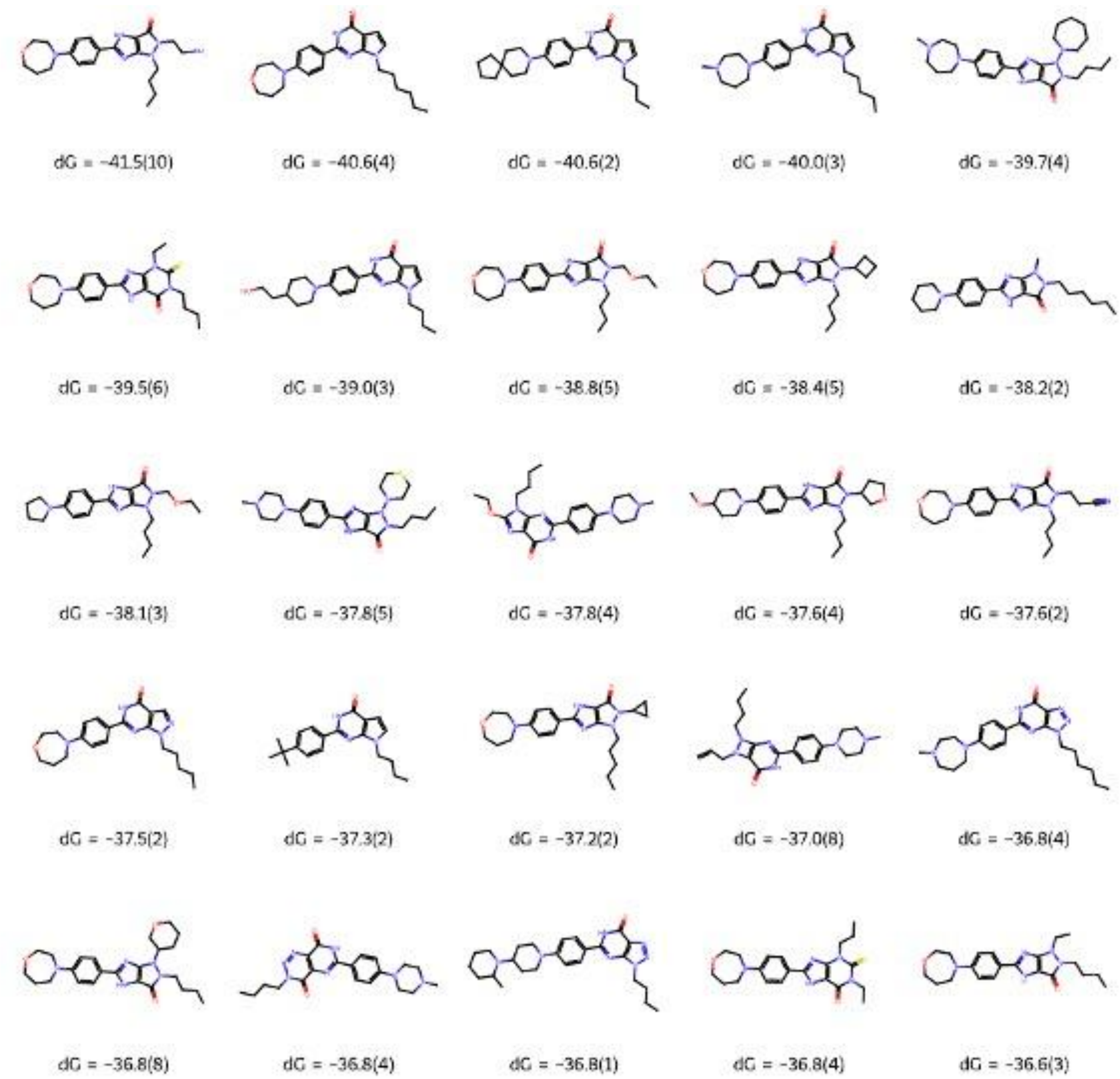

Fig S7: The 25 strongest binding compounds, depicted in order of decreasing binding affinity strength according to ESMACS for TNKS2, found with M1+RA synthesis scoring at the end of GAL. Binding free energies are given in kcal/mol. Statistical errors are given in parenthesis.

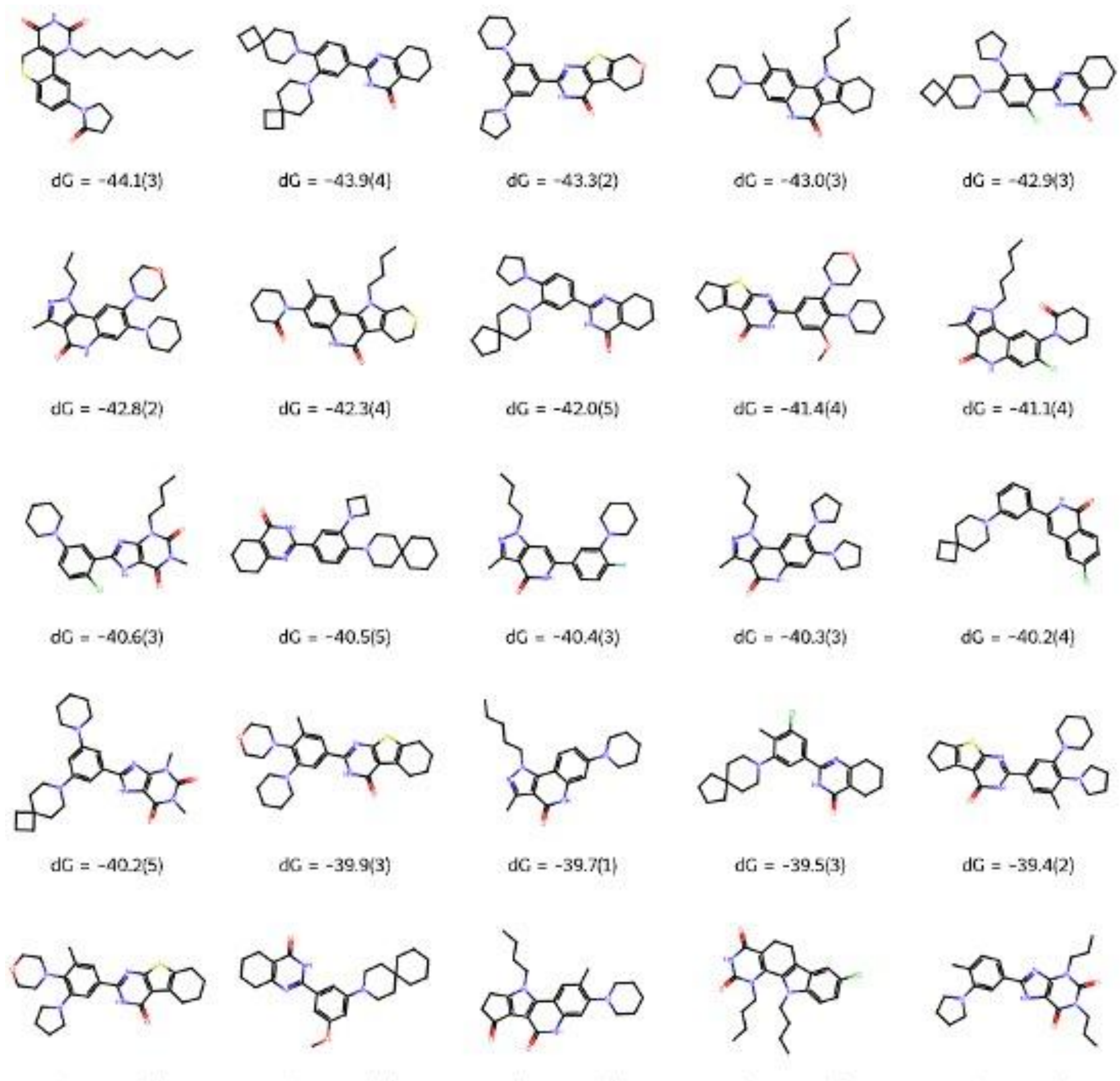

Fig S8: The 25 strongest binding compounds, depicted in order of decreasing binding affinity strength according to ESMACS for TNKS2, found with CAZP synthesis scoring at the end of GAL. Binding free energies are given in kcal/mol. Statistical errors are given in parenthesis.

- In Figures S9-S10 the performance of the GAL surrogate model for 3CL^{pro} is shown for all performed iteration steps to supplement Figure 9 in section 3.3.1.

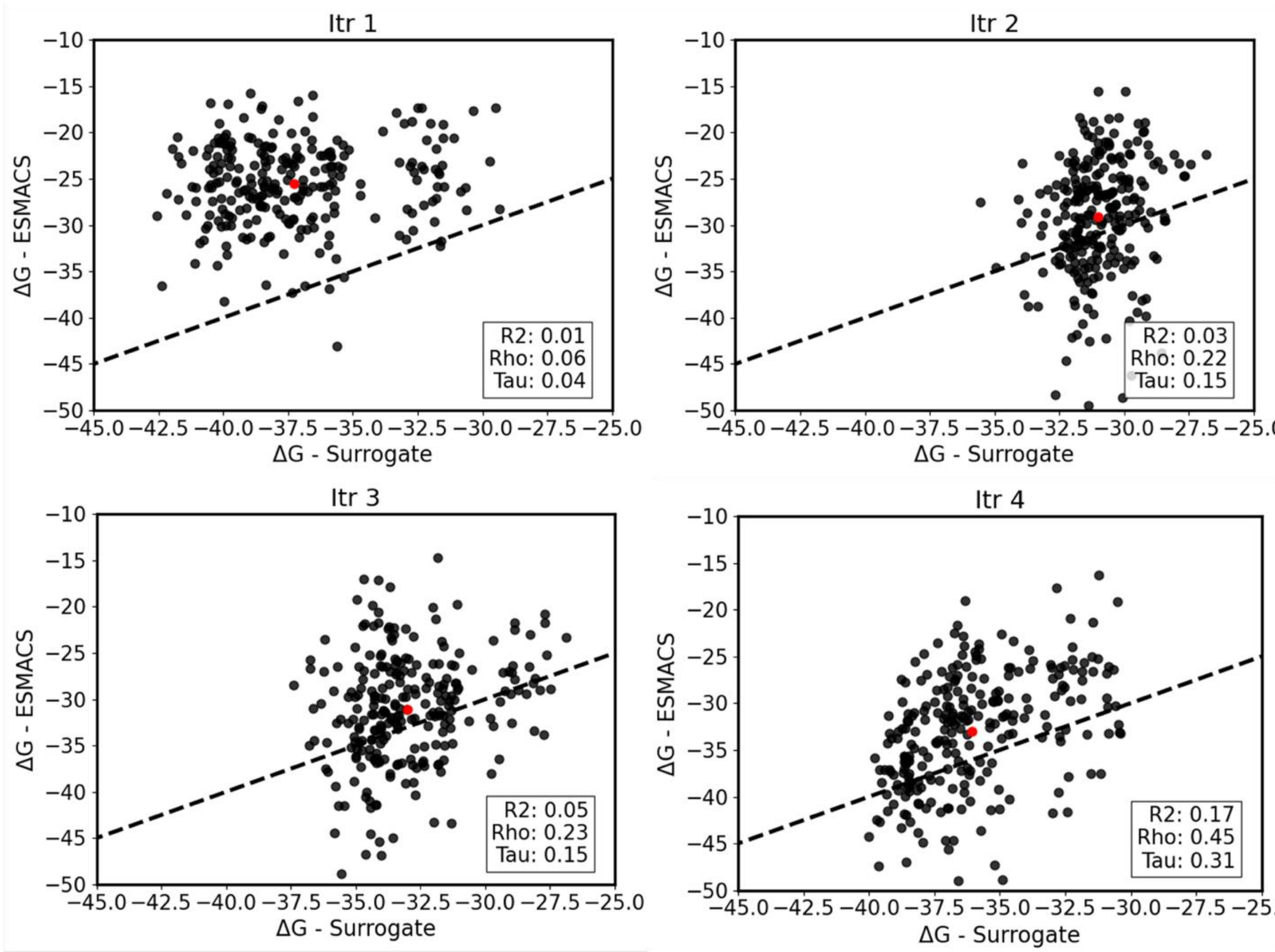

Fig S9: Comparison of ΔG_{bind} predicted by the surrogate model with predictions from the ESMACS oracle for iteration steps 1 to 4 of the AL for 3CL^{pro}. No synthesis scores were used during GAL to provide a reference. The R^2 coefficient, Spearman's ρ, and Kendall's τ are given for individual iterations in the insets. Average values of ΔG from surrogate and ESMACS are indicated with a red dot. All energies are given in kcal/mol.

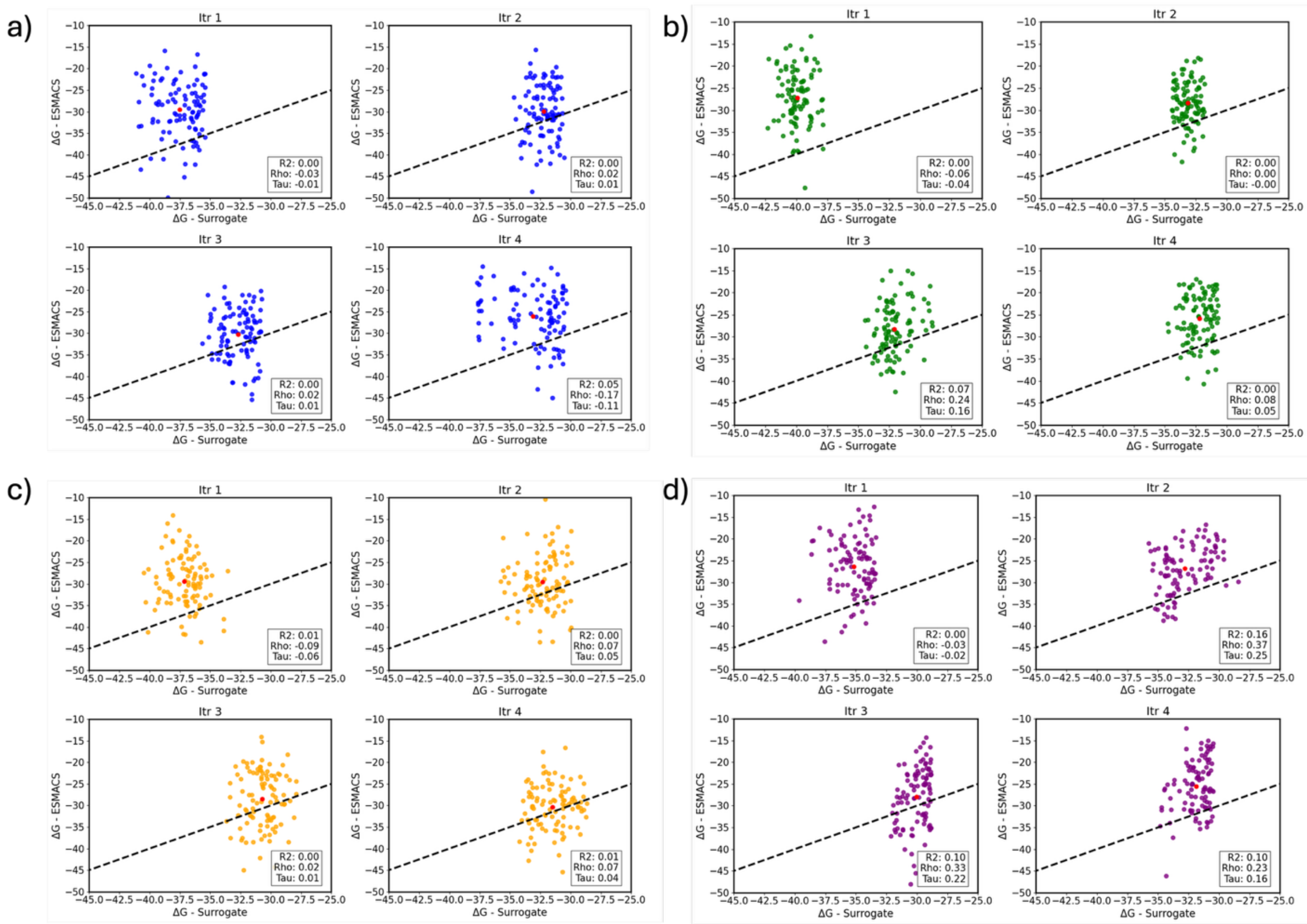

Fig S10: Comparison of ΔG_{bind} predicted by the surrogate model with predictions from the ESMACS oracle for iteration steps 1 to 4 of GAL for $3CL^{pro}$. SYNSs M1 (a), RA (b), RA+M1 (c) and CAZP (d) were used during GAL. The R^2 coefficient, Spearman's ρ, and Kendall's τ are given for individual iterations in the inset legends. Average values of ΔG from surrogate and ESMACS are indicated with a red dot. All energies are given in kcal/mol.

In Figure S11, the distributions of achieved synthesizability scores are shown in case of 3CL^{pro} for all iteration steps and different scoring schemes to support the discussion in section 3.3.3.

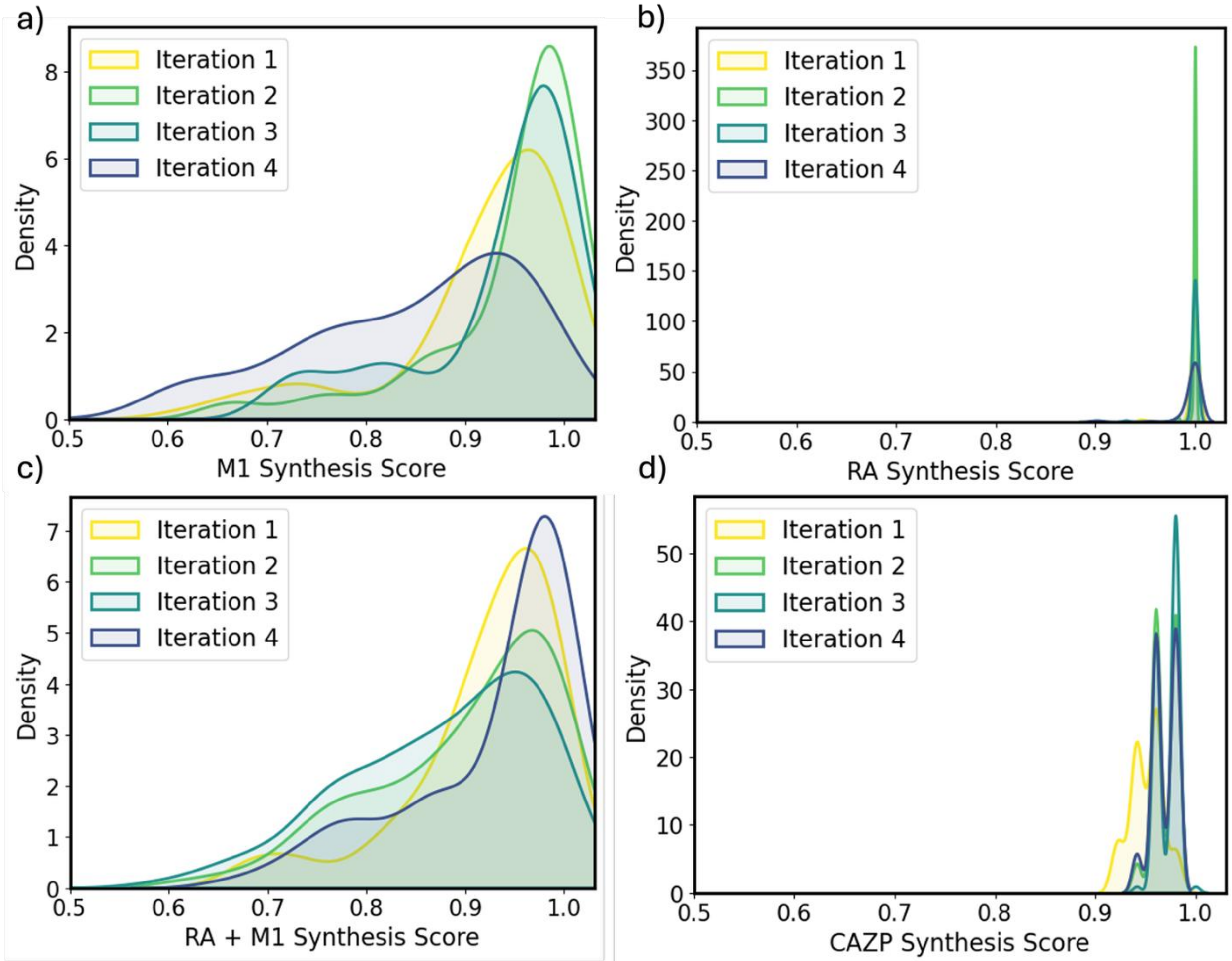

Fig S11: Distribution of SYNS of compounds evaluated with ESMACS after each GAL iteration for 3CL^{pro} with scoring methods M1 (a), RA (b), RA+M1 (c) and CAZP (d) methods.

In Figures S12-S16, all 25 best binding structures, according to ESMACS results, are shown for 3CL^{pro}, after the last GAL iteration step and for different synthesis scoring schemes. These figures provide additional structures to Fig 13 in section 3.3.5 to demonstrate structural variety.

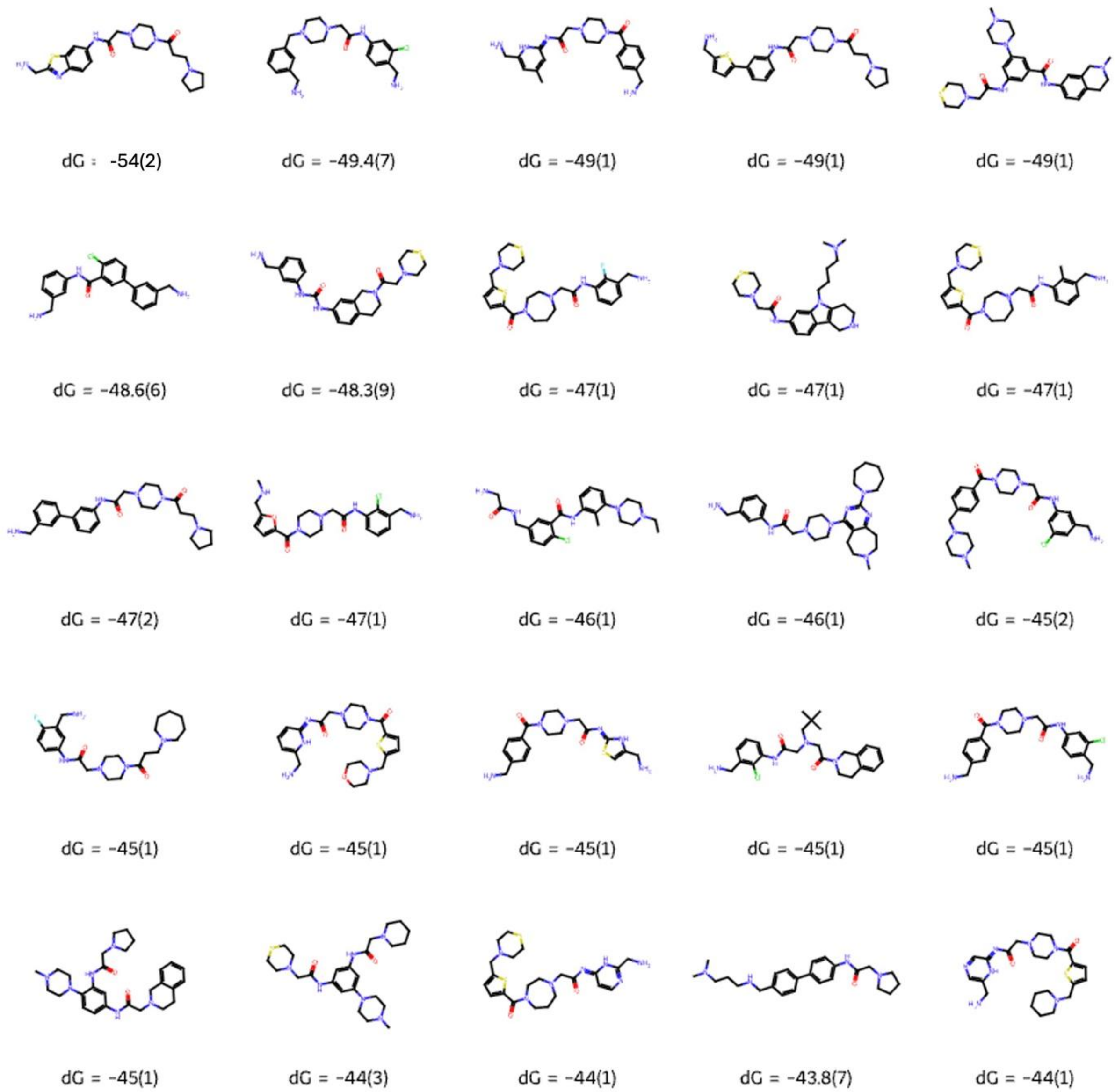

Fig S12: The 25 strongest binding compounds, depicted in order of decreasing binding affinity strength according to ESMACS for 3CL^{pro} at the end of the AL process, when no SYNS was used. Binding free energies are given in kcal/mol. Statistical errors are given in parenthesis.

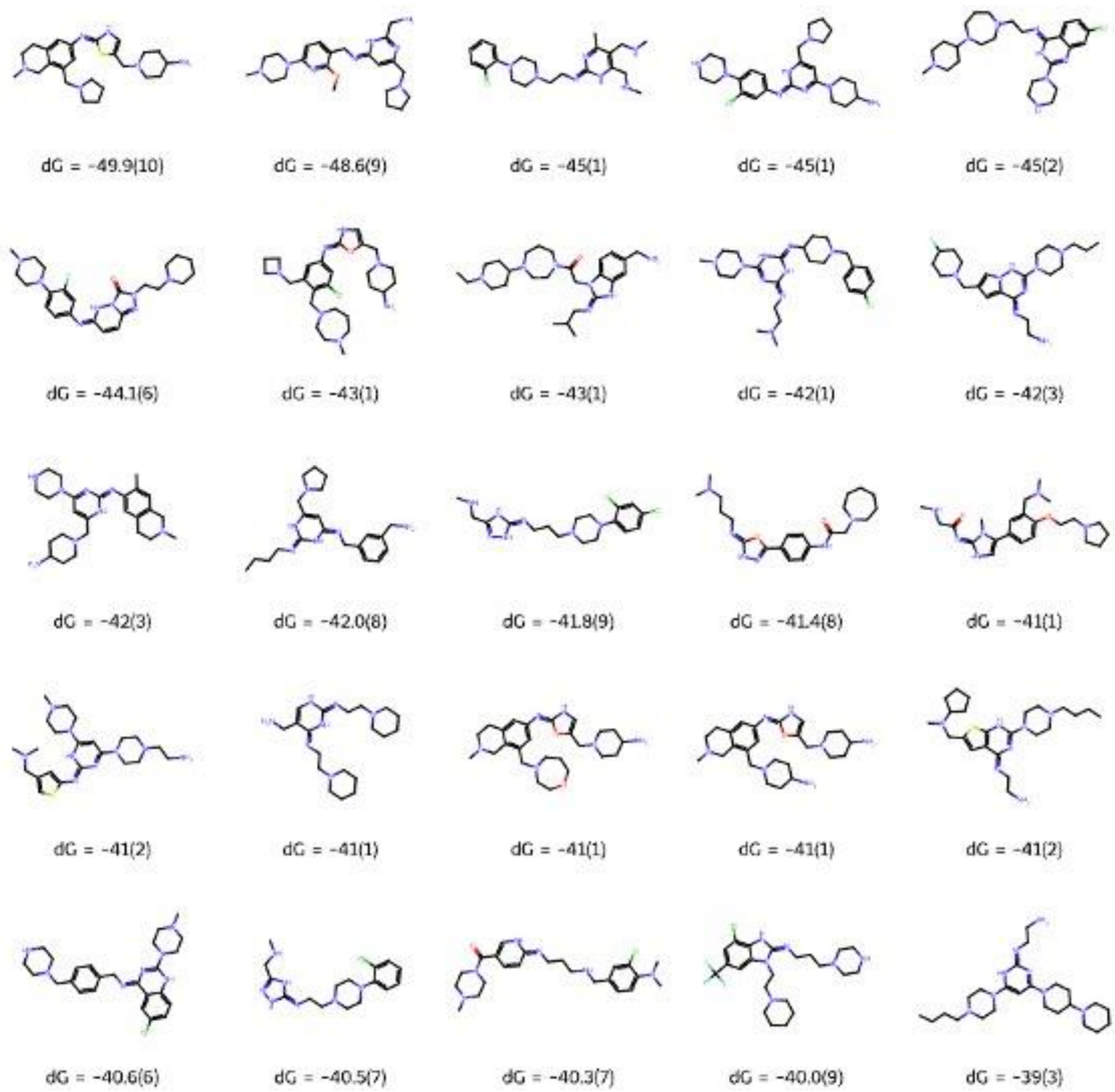

Fig S13: The 25 strongest binding compounds, depicted in order of decreasing binding affinity strength according to ESMACS for 3CL^{pro}, found with M1 synthesis scoring at the end of the AL process. Binding free energies are given in kcal/mol. Statistical errors are given in parenthesis.

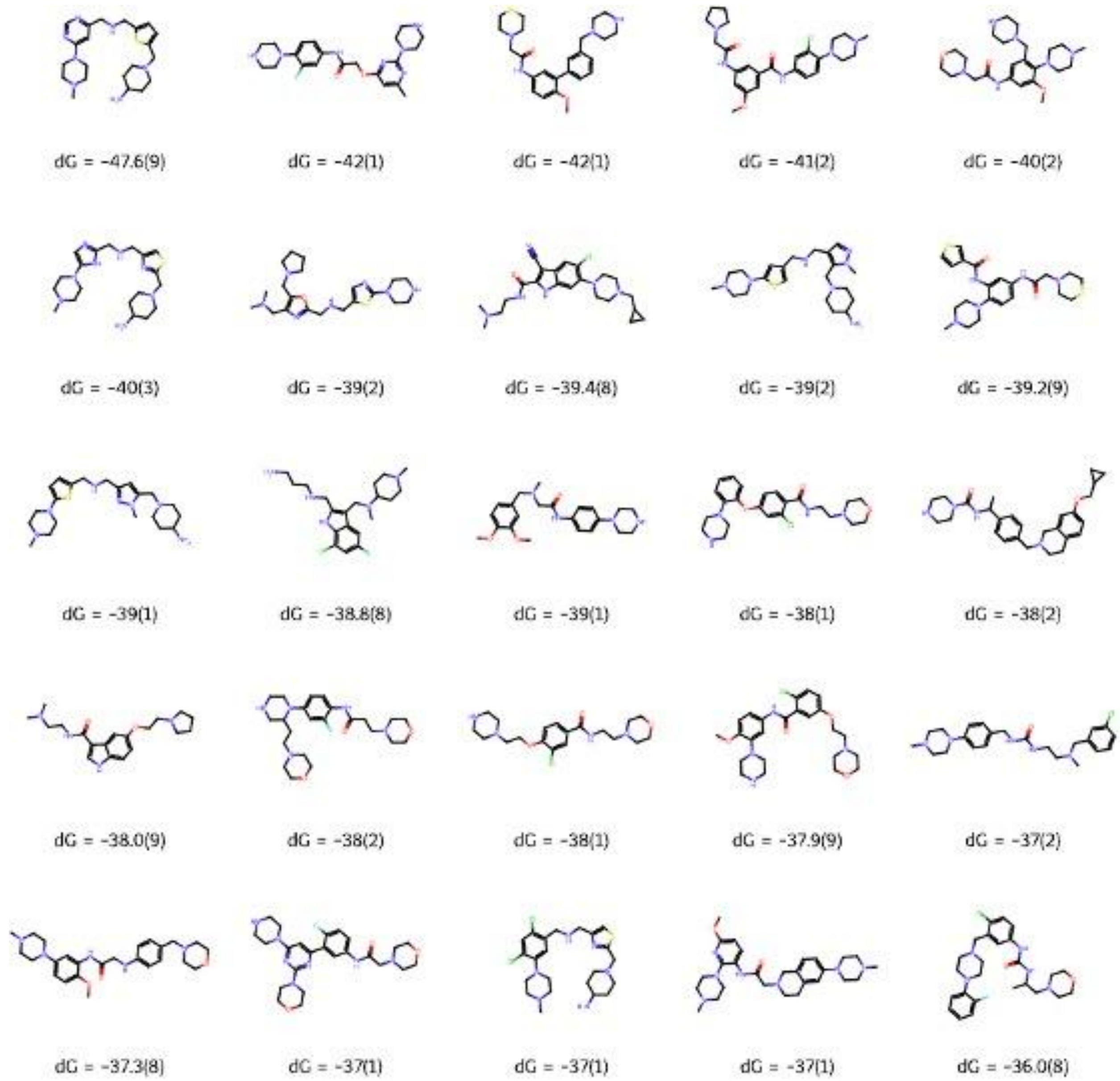

Fig S14: The 25 strongest binding compounds, depicted in order of decreasing binding affinity strength according to ESMACS for $3CL^{pro}$, found with RA synthesis scoring at the end of the AL process. Binding free energies are given in kcal/mol. Statistical errors are given in parenthesis.

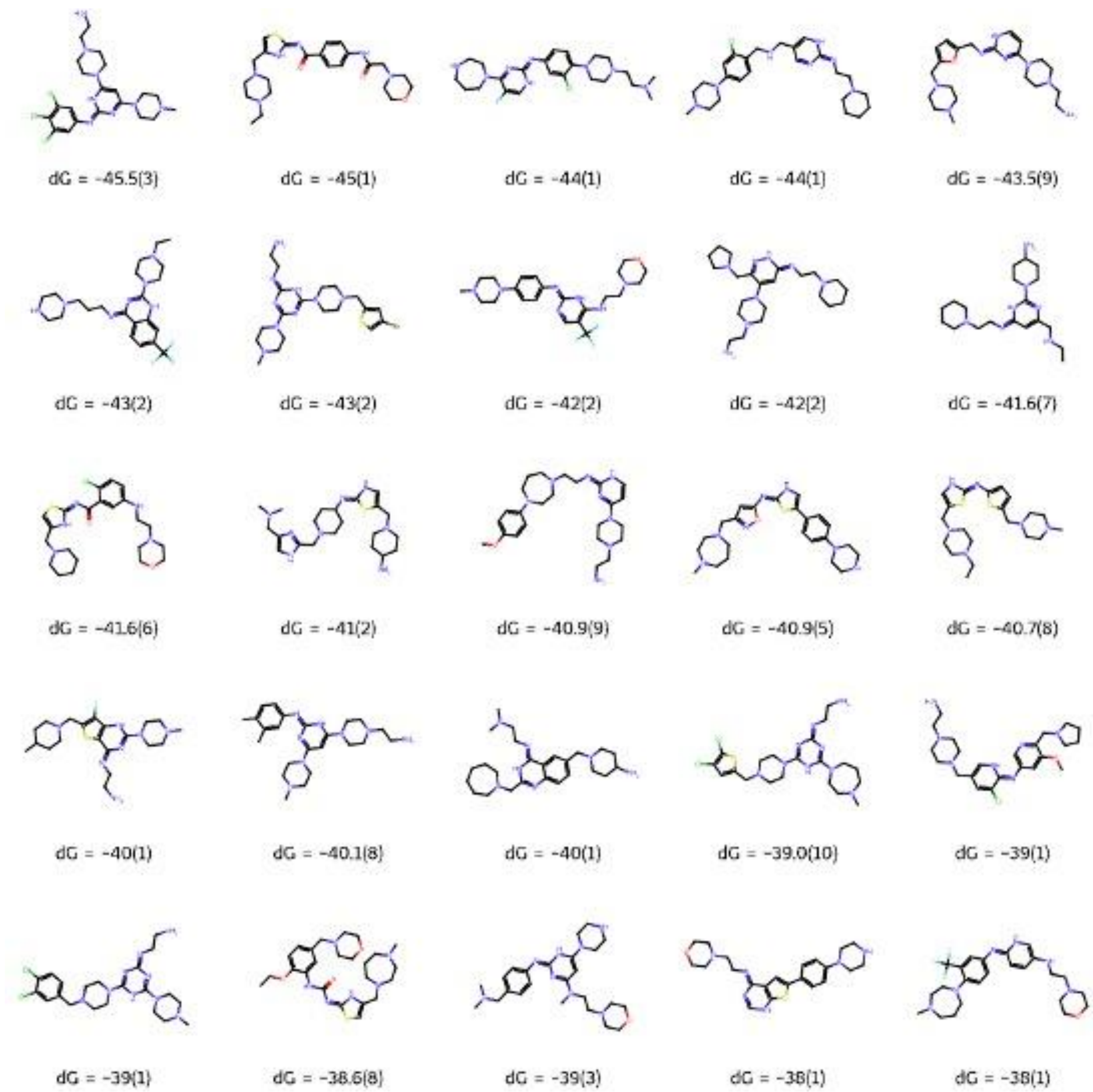

Fig S15: The 25 strongest binding compounds, depicted in order of decreasing binding affinity strength according to ESMACS for $3CL^{pro}$, found with RA+M1 synthesis scoring at the end of the AL process. Binding free energies are given in kcal/mol. Statistical errors are given in parenthesis.

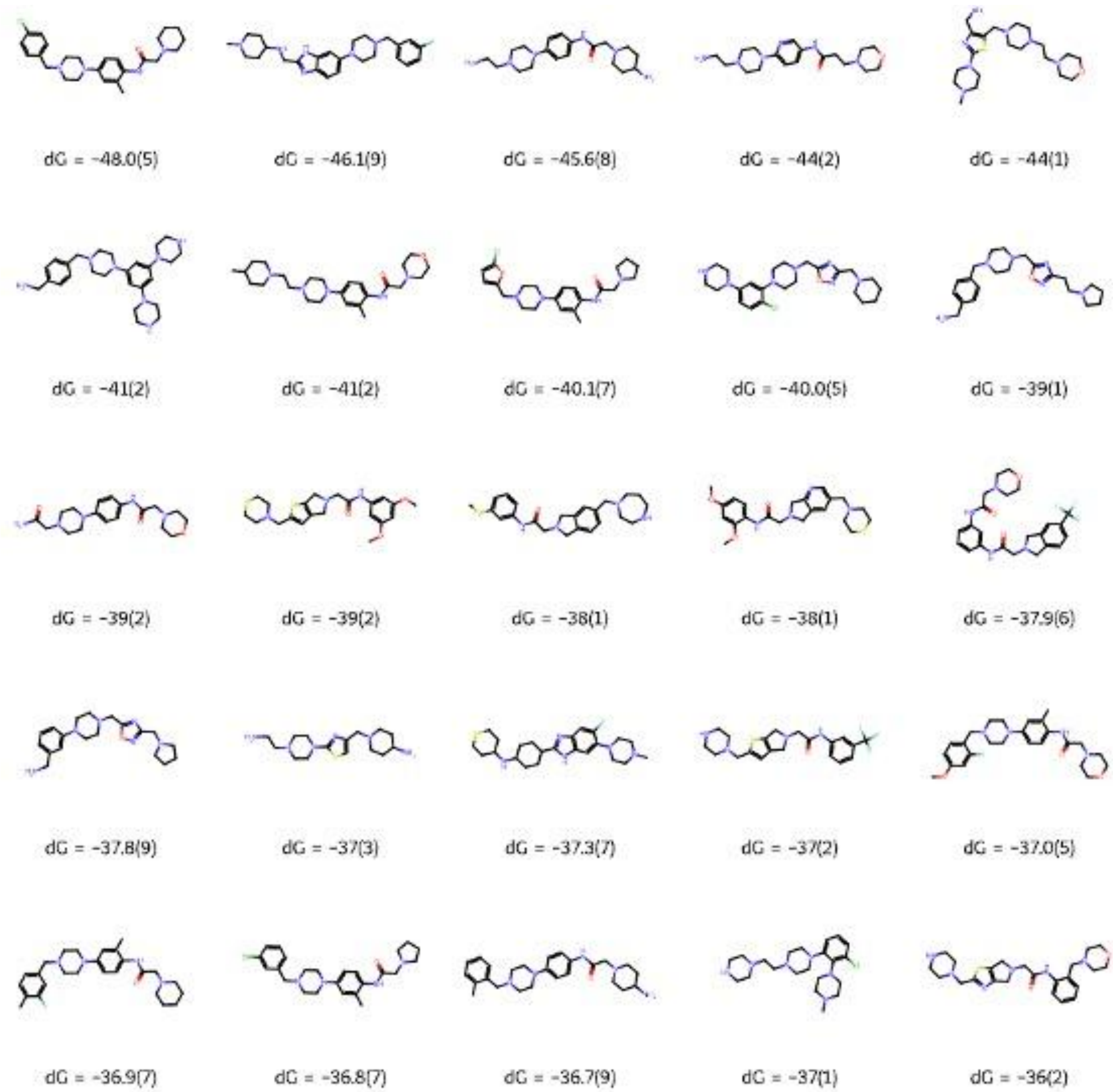

Fig S16: The 25 strongest binding compounds, depicted in order of decreasing binding affinity strength according to ESMACS for $3CL^{pro}$, found with CAZP synthesis scoring at the end of the AL process. Binding free energies are given in kcal/mol. Statistical errors are given in parenthesis.
